# The Role of the MYC/miR-150/MYB/ZDHHC11 Network in Hodgkin Lymphoma and Diffuse Large B-Cell Lymphoma

**DOI:** 10.3390/genes13020227

**Published:** 2022-01-25

**Authors:** Lotteke J. Y. M. Ziel-Swier, Yichen Liu, Annika Seitz, Debora de Jong, Jasper Koerts, Bea Rutgers, Rianne Veenstra, Fazlyn R. Abdul Razak, Agnieszka Dzikiewicz-Krawczyk, Anke van den Berg, Joost Kluiver

**Affiliations:** 1 Department of Pathology and Medical Biology, University Medical Center Groningen, University of Groningen, 9700 RB Groningen, The Netherlands; l.j.y.m.swier@umcg.nl (L.J.Y.M.Z.-S.); y.liu02@umcg.nl (Y.L.); a.seitz@umcg.nl (A.S.); d.de.jong03@umcg.nl (D.d.J.); j.a.koerts@umcg.nl (J.K.); b.rutgers@umcg.nl (B.R.); rianneveenstra@icloud.com (R.V.); a.van.den.berg01@umcg.nl (A.v.d.B.); 2 Neogenix Laboratoire SDB BHD, Petaling Jaya 47301, Malaysia; reeny@neogenix.org; 3 Institute of Human Genetics, Polish Academy of Sciences, 60-479 Poznan, Poland; krawczyk@man.poznan.pl

**Keywords:** miR-150, ZDHHC11, B-cell lymphoma

## Abstract

We previously described involvement of the MYC/miR-150/MYB/ZDHHC11 network in the growth of Burkitt lymphoma (BL) cells. Here we studied the relevance of this network in the two other B-cell lymphomas: Hodgkin lymphoma (HL) and diffuse large B-cell lymphoma (DLBCL). Expression levels of the network components were assessed at the RNA and protein level. The effect of modulating levels of the network components on cell growth was determined through GFP competition assay. AGO2-RNA immunoprecipitation was performed to validate targeting by miR-150. Expression levels of MYC, MYB and ZDHHC11 were increased, while miR-150 levels were decreased similar to the pattern observed in BL. The knockdown of MYC, MYB and ZDHHC11 decreased the growth of HL and DLBCL cells. In contrast, overexpression of miR-150 did not induce clear phenotypes in HL, and limited the effects in DLBCL. This could not be explained by the differences in overexpression levels. Furthermore, we showed that in HL, ZDHHC11 and MYB are efficiently targeted by miR-150. To conclude, MYC, MYB and ZDHHC11 are critical for the growth of HL and DLBCL cells consistent with the role observed in BL cells, while low endogenous miR-150 levels appeared to be less critical for the growth of HL and DLBCL cells despite the effective targeting of ZDHHC11 and MYB.

## 1. Introduction

Burkitt lymphoma (BL) is a highly aggressive germinal center (GC) B-cell derived lymphoma subtype occurring most commonly in children. The hallmark of BL is the translocation of the MYC gene locus to one of the immunoglobulin gene loci, which results in high expression of the oncogenic transcription factor MYC [1]. MYC regulates the expression of many protein-coding and non-coding genes [2,3,4,5,6]. One of the non-coding targets is the MYC-repressed microRNA-150 (miR-150), an important tumor suppressor in lymphoma [6,7,8]. Two validated targets of miR-150 in B-cells are the transcription factor MYB [9,10] and ZDHHC11 [11]. The ZDHHC11 gene encodes a protein that belongs to the zinc finger DHHC domain-containing (ZDHHC) enzyme (S-palmitoyltransferase) family of 24 members. ZDHHC proteins affect the stability, localization and function of other proteins through palmitoylation [12]. Besides a protein-coding transcript, two other transcripts have been identified from the ZDHHC11 locus in BL: a linear long non-coding (lnc) and a circular non-coding (circ) RNA transcript. All three transcripts contain up to 18 binding sites for miR-150 and were shown to bind miR-150. In BL, MYC, MYB and ZDHHC11 show increased levels and miR-150 decreased levels compared to normal germinal center (GC) B-cells. The knockdown of MYB, MYC and all ZDHHC11 transcripts strongly inhibited cell growth in BL, while overexpression of miR-150 inhibited BL growth by the targeting of MYB and ZDHHC11 [11]. Here we studied the relevance of the MYC/miR-150/MYB/ZDHHC11 network in two other GC B-cell derived lymphomas, i.e., Hodgkin lymphoma (HL) and diffuse large B-cell lymphoma (DLBCL).

## 2. Materials and Methods

### 2.1. Culture of Cell Lines and Sorting of GC B-Cells

Cell lines used throughout the manuscript are listed in Supplementary Table S1, including their origin and culture conditions. Cell lines were either cultured in RPMI 1640 (Gibco, Waltham, MA, USA; Lonza BioWhittaker, Walkersville, MD, USA in case of ST486), McCoys5A (Lonza BioWhittaker, Walkersville, MD, USA) or DMEM (Lonza BioWhittaker, Walkersville, MD, USA), supplemented with 10–20% fetal bovine serum (Cambrex Biosciences, Walkersville, MD, USA). All media were supplemented with 100 units/mL of penicillin, 100 µg/mL of streptomycin and 2 mM of glutamine (Cambrex Biosciences, Walkersville, MD, USA). The origin of the cell lines was confirmed with STR DNA analyses on a regular basis and mycoplasma tests were performed to exclude contamination. The cultures were maintained at 37 °C in a humidified air atmosphere supplemented with 5% CO_2_.

GCB-cells (defined as CD20+IgD−CD38+) were sorted from routinely removed tonsil specimens as described previously [13]. Written permission for the use of the tonsil tissues to isolate GCB-cells was obtained from the parents of the children. The study protocol was consistent with international ethical guidelines (the Declaration of Helsinki and the International Conference on Harmonization Guidelines for Good Clinical Practice).

### 2.2. DNA Constructs and Viral Transfection

Lentiviral shRNA constructs targeting all ZDHHC11 transcripts, and MYC and MYB were described and tested previously [11]. Non-targeting control shRNA constructs were consistently used as negative controls. Lentiviral vectors for miR-150 overexpression and empty vector controls were described previously [14]. Lentiviral transfections were performed as previously described [15,16], aiming for an infection percentage of 10–50% to follow the effect on cell growth over time. To validate miRNA overexpression levels, we infected cells aiming at an infection percentage of >70%. In case of low GFP percentages, infected cells were puromycin selected for 1–2 weeks. Cells were harvested and stored at −20 °C until further processing. 

### 2.3. GFP Competition Assay 

The percentages of GFP positive cells were measured on the BD Accuri™ C6 Plus Cell Analyzer (BD Biosciences, Franklin Lakes, NJ, USA) at day 4 post-transfection and monitored tri-weekly for three weeks. Data were analyzed using FlowJo software (version 10, Treestar, Ashland, OR, USA). To determine the effect on cell growth, the percentage of GFP positive cells at day 4, 6 or 8 (depending on the time reaching the maximal GFP percentage which varied per construct and cell line) was set to 100% and the fold difference relative to this starting point was calculated for each time point. To determine significant differences in the GFP assays, we used mixed model analysis as described previously [11]. 

### 2.4. RNA Isolation and RT-qPCR 

Total RNA was isolated using the miRNeasy mini or micro kit (Qiagen, Germantown, MD, USA) in combination with Phase Lock Gel Heavy tubes (5 Prime, Hilden, Germany). The primers used for the quantification of the transcript levels are shown in Supplementary Table S2. For the quantification of the circular ZDHHC11 transcript, an input of 10 ng of cDNA was used, and for the other transcripts, we used an input of 1 ng in a final volume of 10 µL. The synthesis of cDNA and amplification were performed as described previously [11]. Expression levels were normalized to U6 and expressed as 2-delta Cp. For the quantification of miRNA overexpression, multiplex miRNA-specific cDNA synthesis and RT-qPCR was performed using the TaqMan MicroRNA Reverse Transcription Kit and TaqMan MicroRNA Assays (both Applied Biosystems, Waltham, MA, USA) according to the manufacturer’s protocol. The MicroRNA Assay IDs were as follows: 000473 (miR-150-5p), 000391 (miR-16-5p), 002308 (miR-17-5p) and 001006 (RUN48). MiRNA levels were normalized to RNU48 and expressed as 2-delta Cp.

### 2.5. AGO2 RNA Immunoprecipitation

Immunoprecipitation of AGO2-containing RISC complexes was performed as described previously [17] with some small modifications. EZview protein G beads (Merck Life Science NV, Amsterdam, the Netherlands) were pre-blocked using 5% BSA and 2 µg/µL salmon sperm sonicated ssDNA (Merck Life Science NV). Lysates from 30–40 million L428 and SUPHD1 cells were incubated with anti-AGO2 antibody (clone 2E12-1C9, Abnova, Taipei City, Taiwan) coated beads overnight at 4 °C while gently rocking. As a negative control, cells were also incubated with anti-IgG antibody (Millipore BV, Amsterdam, The Netherlands) coated beads.

Total RNA of the total fractions (TF) and immunoprecipitated (IP) fractions were isolated as described above and used for analysis by microarray using an Agilent SurePrint G3 Human GE 8x60K microarray (Agilent ID 72363) as described previously [11]. The microarray data were deposited in the Gene Expression Omnibus (GSE188310). Genes were identified as specific targets of miR-150 when the IP/TF ratio in the HL cells transfected with the miR-150 overexpression vector was ≥twofold higher than the IP/TF ratio in the HL cells transfected with the empty overexpression vector. For comparison to the miR-150-specific targets previously identified in BL [11], we focused on genes for which probes were present on both microarrays. 

### 2.6. Western Blot 

Whole cell protein lysates were prepared using cell lysis buffer (Cell Signaling) supplemented with 1 mM PMSF. The total protein concentration was measured using the Pierce BCA Protein Assay kit (Thermo Fisher Scientific, Waltham, MA, USA). After denaturing by boiling for 5 min at 100 °C in loading buffer, proteins were separated by SDS-PAGE using 10% acrylamide gels. Next, the proteins were transferred to nitrocellulose membranes using the Bio-Rad Wet electroblotting system (Life Science Technologies). Antibodies were diluted in 5% milk in Tris-buffered saline + Tween-20. The primary, secondary and tertiary antibody solutions used are listed in Supplementary Table S3. For detection of GAPDH, Pierce™ ECL Western blotting Substrate (Thermo Fisher Scientific, USA) was applied to the blots. SuperSignal West Pico Chemiluminescent Substrate (Thermo Fisher Scientific, USA) was applied to the blots for detection of the other proteins. Protein bands were visualized with a ChemiDoc MP scanner and bands were quantified using the Image Lab 6.0 software (both BioRad, Veenendaal, The Netherlands). Protein levels were normalized to GAPDH or to the total amount of protein loaded on gel.

### 2.7. Analysis of Deposited Microarray and RNA-seq Data 

For cHL, normalized and log2 transformed microarray expression data of probes detecting all three ZDHHC11 transcripts (Affymetrix probe set 232417_x_at) were retrieved from the GEO accession viewer (http://www.ncbi.nlm.nih.gov/geo/ accessed on 23 April 2021; GSE39132 (5 CD77+ GC B-cell samples) and GSE39133 (29 HRS cell samples) for the Steidl cohort; GSE12453 (10 CD77-/+ GC B-cell samples and 12 HRS cell samples) for the Tiacci cohort; GSE12453 (12 HRS cell samples) and GSE83441 (5 CD30+ GC B-cell samples) for the Weniger cohort) [18,19,20].

For DLBCL, TMM-normalized RNA-seq expression data for ZDHHC11 mRNA was selected from the Scott & Ennishi cohort (*n* = 283) [21,22]. Assignment into cell-of-origin groups was performed using Lymph2Cx digital gene expression profiling. Normalized and log2 transformed microarray expression data of the Visco cohort (*n* = 489) for all ZDHHC11 transcripts using the Affymetrix probe as described above was obtained from the NCBI Gene Expression Omnibus (GEO accession number GSE31312) via the R2: Genomics Analysis and Visualisation Platform (http://r2.amc.nl accessed on 23 April 2021) [23]. Assignment into cell-of-origin groups was performed using TMA immunohistochemistry.

### 2.8. Statistical Analysis

GraphPad Prism 6 (GraphPad Software Inc., La Jolla, CA, USA) was used to perform the statistical analysis. Data was represented as mean with SD, median or median with interquartile range. Statistical analysis of the differences between median was calculated with the one-sided Mann-Whitney test for the comparison of two groups, or the Kruskal-Wallis test with Dunn’s post-hoc test when more than two groups were compared.

## 3. Results

### 3.1. Expression of Network Components in B-Cell Lymphoma

**Figure 1:**
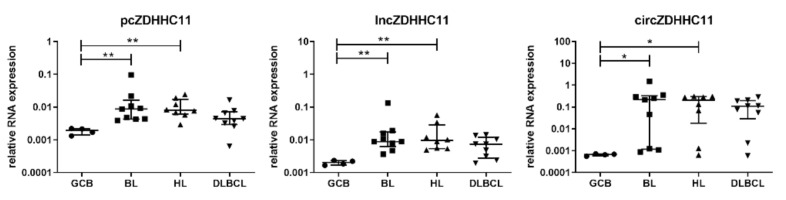
Expression pattern of the three ZDHHC11 transcripts in B-cell lymphoma cell lines and germinal center B-cells (GCB). Relative RNA expression of the protein coding ZDHHC11 (pcZDHHC11), linear non-coding ZDHHC11 (lncZDHHC11) and circular ZDHHC11 (circZDHHC11) in GC B-cells sorted from tonsils (*n* = 4), Burkitt lymphoma (BL, *n* = 9), Hodgkin lymphoma (HL, *n* = 8) and diffuse large B-cell lymphoma (DLBCL, *n* = 9) cell lines were determined by RT-qPCR. The expression was normalized to U6. Kruskal-Wallis test with Dunn’s post-hoc tests were used to assess significance; * *p* < 0.05, ** *p* < 0.01, data are represented as median with interquartile range. For better visualization in the figures, expression levels of circZDHHC11 were multiplied with 1000.

In our previous study, we already showed similar expression patterns for the network components in HL and DLBCL as compared to BL, i.e., increased MYB, MYC and ZDHHC11 levels and decreased miR-150 levels compared to GC B-cells [11]. As a next step, we tested the expression levels of the three individual ZDHHC11 transcripts in a set of 30 B-cell lymphoma cell lines and GC B-cells. The levels of each of the three distinct ZDHHC11 transcripts were increased in BL, HL and DLBCL cell lines compared to GC B-cells (Figure 1), with significant differences for BL and HL in comparison to GC B-cells. The expression patterns were similar to the results observed for the general primer set targeting all three ZDHHC11 transcripts (Figure S1). For DLBCL, we observed the same trend, albeit less pronounced and not significantly different. Expression levels for each of the ZDHHC11 transcripts were consistently low in GC B-cells and increased in the cell lines, albeit at variable levels, especially for circZDHHC11. For completeness, we also determined the mRNA expression levels of MYC, MYB and miR-150 in this extended cell line panel (Figure S1). The expression patterns of the other network components also showed the same trend in HL and DLBCL as observed for BL in line with our previous results [11].

**Figure 2:**
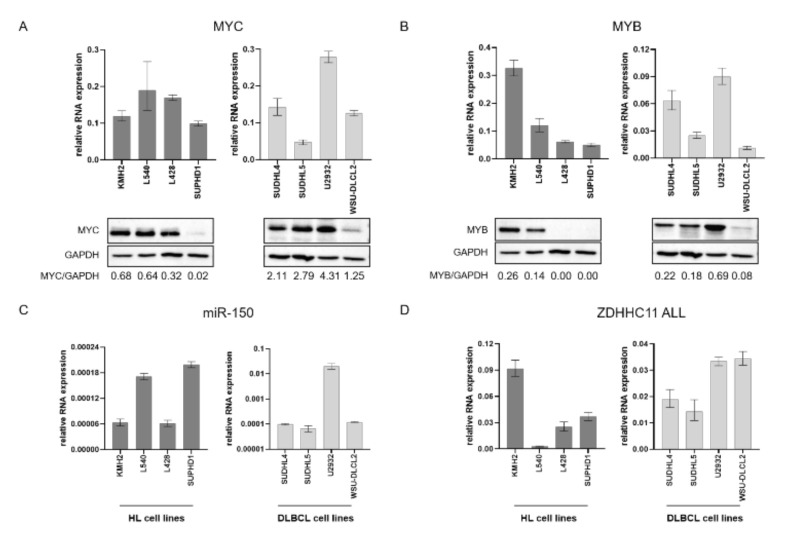
MYC and MYB protein and MYC, MYB, miR150 and ZDHCC11 transcript levels in HL and DLBCL cell lines. Relative RNA expression (upper panels) of (**A**) MYC, (**B**) MYB, (**C**) miR150, and (**D**) ZDHHC11 all transcripts (ZDHHC11 ALL) in 4 HL (KMH2, L540, L428, SUPHD1) and 4 DLBCL (SUDHL4, SUDHL5, U2932, WSUDLCL2) cell lines. The RNA levels were normalized to U6. Kruskal-Wallis test with Dunn’s post-hoc tests were used to assess significant differences; data are represented as median with interquartile range. Western blots (lower panels) showing (**A**) MYC and (**B**) MYB protein levels. GAPDH was used as a loading control and for quantification. Fold differences relative to the ST486 BL cell line are shown below the blots (see full blot, including ST486 in Figure S2 for uncropped blots).

Next, we tested the presence of MYC and MYB protein in a subset of the HL and DLBCL cell lines also used for functional follow-up experiments (Figure 2 and Figure S2). MYC protein levels were higher in KMH2, L428 and L540 HL cell lines as compared to SUPHD1. In DLBCL cell lines, SUDHL4, SUDHL5 and U2932 had higher levels compared to WSU-DLCL2. MYB protein was detected in two of the HL cell lines and in all of the DLBCL, with lower levels present again in WSU-DLCL2. The two MYB negative HL cell lines, i.e., L428 and SUPHD1, harbored a nonsense mutation in MYB that led to a truncated MYB protein, which cannot be detected with the antibody raised against the C-terminus of MYB that was used in the WB [24]. Despite testing multiple commercially available ZDHHC11 antibodies, we were unable to reliably detect the ZDHHC11 protein. Overall, the observed expression patterns of the three individual ZDHHC11 transcripts and the other network components, i.e., MYC, MYB and miR-150, in HL and DLBCL cell lines were similar to those observed in BL. No obvious correlation could be observed between MYC mRNA and protein levels in either HL or DLBCL cell lines. MYB mRNA and protein levels did show a moderate correlation in both HL and DLBCL, with exception of the two HL cell lines with a truncating mutation in MYB, resulting in loss of the binding site of the MYB antibody.

To confirm expression of ZDHHC11, i.e., the novel component of the MYC-miR-150-MYB network, in primary HL and DLBCL we analyzed previously published expression profiling studies. The levels of the ZDHHC11 transcripts were moderate to strongly increased in purified primary HRS cells compared to GC B-cells in all three cohorts (Figure S3). A possible explanation for the observed variation in ZDHHC11 increases may be related to the different approaches used to isolate GC B-cells. ZDHHC11 was also expressed in primary DLBCL cases with significantly higher levels in GCB DLBCL compared to ABC DLBCL cases in both cohorts. These data indicate that ZDHHC11 is expressed in primary HRS and DLBCL cells, further underscoring the potential relevance of this network in HL and DLBCL. 

### 3.2. Effect of MYC, MYB, ZDHHC11 and miR-150 on Growth of HL and DLBCL Cell Lines

**Figure 3:**
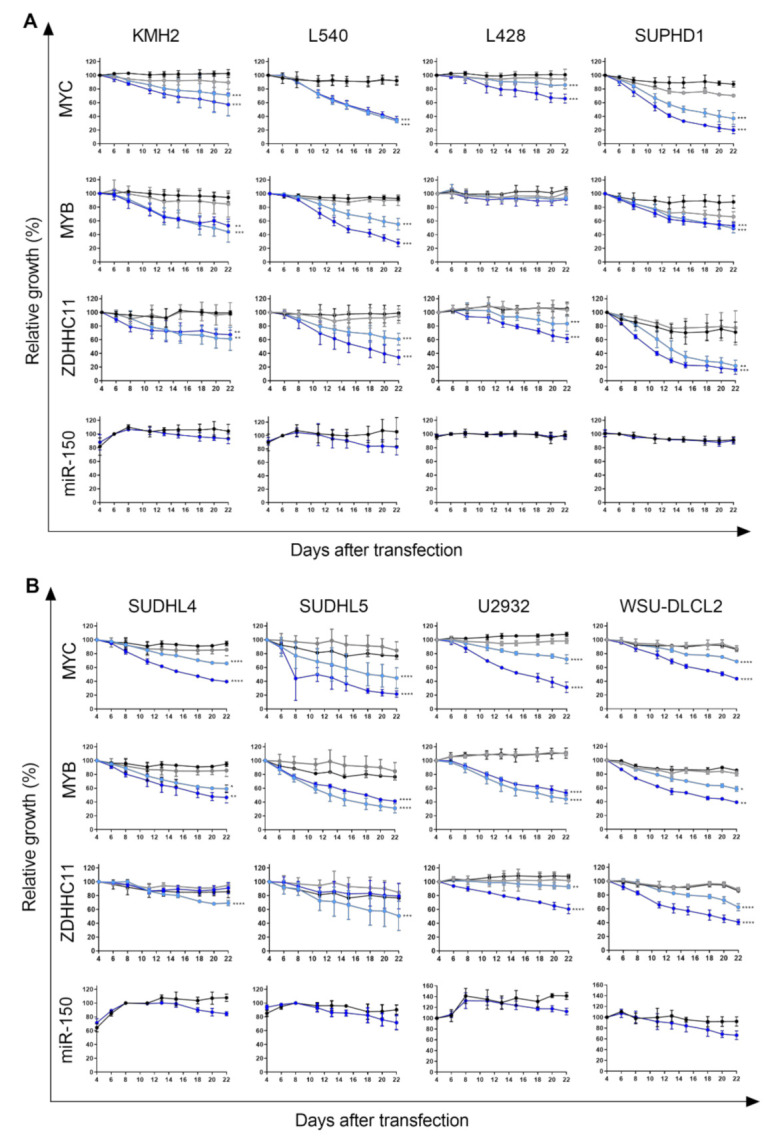
Effect of MYC, MYB and ZDHHC11 knockdown and miR-150 overexpression on growth of HL and DLBCL cell lines. (**A**) HL and (**B**) DLBCL cell lines were infected with a lentivirus carrying a shRNA construct targeting MYC, MYB or ZDHHC11 transcripts (two shRNAs per transcript, shRNA-1 in light and shRNA-2 in dark blue) or shRNA control vectors (black and grey). An overexpression vector was used for miR-150 (dark blue), and an empty overexpression vector as a control (black). The effect on cell growth was assessed by following the percentage of GFP+ cells for three weeks post-transfection (*n* = 3), with the GFP percentage normalized to day four, six or eight after transfection. Mixed model analysis was used to determine significant differences; * *p* < 0.05, ** *p* < 0.01, *** *p* < 0.001, **** *p* < 0.0001.

Next, we tested the effect of MYC, MYB or ZDHHC11 knockdown in HL and DLBCL cell lines. The effectiveness of the shRNAs targeting MYC, MYB and ZDHHC11 transcripts has been shown in our previous publication and were confirmed for MYC and MYB in the L540 HL cell line (Figure S4) [5,11]. The knockdown of MYC revealed a strong negative effect on growth, with a relative decrease in GFP positive cells of more than 40% in most cell lines at day 22 after infection (Figure 3). Two HL cell lines, KMH2 and L428, and one DLBCL cell line, WSU-DLCL2, showed a milder effect on growth upon MYC knockdown with a decrease in GFP of 20–35%. The knockdown of MYB induced a mild effect in the two HL cell lines with nonsense mutations, SUPHD1 (33%) and L428 (12%), while it had a strong inhibiting effect on growth of the other HL and all DLBCL cell lines, with a decrease in GFP of more than 40%. 

The knockdown of all ZDHHC11 transcripts also inhibited growth of HL and DLBCL cell lines, albeit at variable levels ranging from mild to strong effects. In HL, both shRNAs showed a strong phenotype in SUPHD1 and L540, with a reduction of 78% and 40% for shRNA-1 and 82% and 70% for shRNA-2. The effects on growth of KMH2 and L428 cells were milder with 40 and 20% for shRNA-1 and 35 and 40% for shRNA-2. In DLBCL cell lines U2932 and WSU-DLCL2, the reduction in GFP was most prominent for shRNA-2 with 40 and 60%, and moderate for shRNA-1 with 10 and 40%. In the two other DLBCL cell lines, SUDHL4 and SUDHL5, only mild effects were observed for shRNA-1 and no effects for shRNA-2. Thus, similar to BL, the inhibition of all ZDHCC11 transcripts resulted in a decrease in growth in all HL and two of the four DLBCL cell lines, while the knockdown of MYC and MYB decreased cell growth in all cell lines.

To establish an effect of miR-150 overexpression on cell growth, we first tested the overexpression efficiency in all individual cell lines (Figure S5). The fold increase in expression ranged between ~125 and ~1400 and was dependent on the endogenous miR-150 levels. The absolute ectopic expression levels were less variable and in the same range as the levels achieved in BL upon miR-150 overexpression. In our previous work, miR-150 overexpression resulted in a clear negative effect on growth in BL cell lines [11]. Unexpectedly, overexpression of miR-150 induced only very mild effects on growth in two of the four HL cell lines and in all four of the DLBCL cell lines, while no effects were observed in the HL cell lines SUPHD1 and L428. Therefore, in contrast to BL, overexpression of miR-150 has limited to no effect on growth of HL and DLBCL cell lines.

### 3.3. MiR-150 Targets ZDHHC11 and MYB in HL Cells

**Figure 4:**
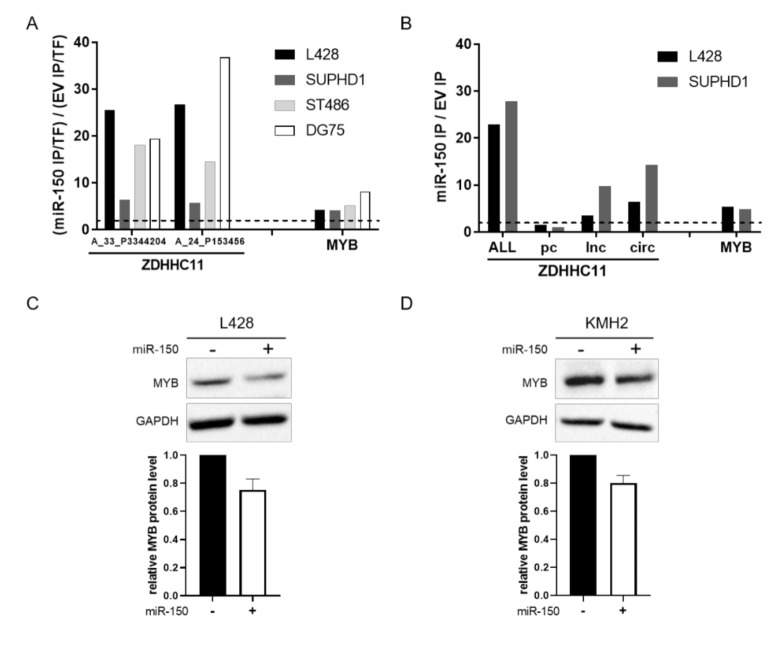
MiR-150 targets ZDHHC11 and MYB in HL cell lines. (**A**) Microarray analysis showing increased enrichment of ZDHHC11 (2 probes) and MYB transcripts in the AGO2 immunoprecipitated (IP) fraction upon miR-150 overexpression in HL (L428 in black, SUPHD1 in dark grey) and BL cell lines (ST486 in light grey, DG75 in white). The dotted line indicates a fold change of 2. (**B**) RT-qPCR analysis showing increased enrichment of ZDHHC11 transcripts (all three transcripts, ALL; protein-coding transcript, pc; linear non-coding transcript, lnc; circular non-coding transcript, circ) and MYB in the AGO2-IP fraction upon miR-150 overexpression in L428 (black) and SUPDH1 (dark grey). The increase in IP enrichment was calculated as the ratio IP/total fraction (TF) fraction in miR-150 overexpressing cells over the ratio IP/TF in empty overexpression vector (EV) cells. Western blots showing MYB levels in the HL cell lines (**C**) L428 and (**D**) KMH2 infected with EV (miR-150−) or miR-150 overexpression vector (miR-150+). The relative expression was normalized to GAPDH (*n* = 2, see also Figure S7).

To explore whether differences in the genes targeted by miR-150 could explain the lack of a phenotype for miR-150 overexpression, we performed AGO2-RNA immunoprecipitation (AGO2-RIP) in two HL cell lines, i.e., L428 and SUPHD1. A good efficiency of the AGO2-RIP was confirmed by showing enrichment of the AGO2 protein by Western blot as well as by enrichment of miR-150, miR-16 and miR-17 by RT-qPCR in the IP fractions (Figure S6). The numbers of IP enriched probes upon miR-150 overexpression were 225 for L428, 46 for SUPHD1, 65 for ST486 and 27 for DG75. Similar to the results in BL, we also showed a clear enrichment of ZDHHC11 and MYB probes upon miR-150 overexpression in the HL cell lines (Figure 4A and Table S4). This indicated that upon miR-150 overexpression, ZDHHC11 and MYB transcripts are targeted by miR-150 in HL cells. In a more detailed analysis of the AGO2-IP fraction by RT-qPCR, we showed that the three individual transcripts of ZDHHC11 were all enriched in the HL cell lines upon miR-150 overexpression (Figure 4B). In line with our results in BL, the circZDHHC11 transcript was the most enriched transcript in both cell lines. The fold enrichment of the pcZDHHC11 transcript was less than 2: making it lower than the enrichment of the two other ZDHHC11 transcripts and suggesting a less effective interaction with miR-150. Consistent with the findings in BL, we also observed a decrease in MYB protein levels upon miR-150 overexpression in KMH2 and L428 supporting effective targeting by miR-150 (Figure 4C,D and Figure S7). Comparison of the other miR-150 target genes identified in the AGO2-RIP experiments in BL and HL revealed no obvious differences between BL and HL that might explain the lack of a phenotype in HL (Figure S8). In ST486, we identified the previously confirmed miR-150 target EGR2, while this gene was not AGO2-IP enriched in the other cell lines. Thus, our data indicate that although miR-150 can effectively bind to the transcripts of ZDHHC11 and MYB, it does not induce a clear phenotype in HL.

## 4. Discussion

The MYC/miR-150/MYB/ZDHHC11 network was previously established in BL and was shown to have a cell growth regulatory effect [11]. Here we explored the role of the network in HL and DLBCL. The expression of the four components of the network showed the same pattern in HL and DLBCL as compared to BL. Analysis of the three individual ZDHHC11 products, the protein coding, the long non-coding and the circular ZDHHC11 transcripts, in HL and DLBCL also showed patterns that were consistent with those observed in BL. Despite having similar expression patterns of the network components and similar effects upon knockdown of MYC, MYB and ZDHHC11 on cell growth, miR-150 overexpression induced no or limited effects on growth of four HL and four DLBCL cell lines. 

Next, we explored why miR-150 overexpression did not affect the growth of HL and DLBCL cell lines. A first potential explanation might be that the achieved miR-150 levels are insufficient to induce a phenotype. A comparison of miR-150 overexpression efficiencies of HL and DLBCL with BL cell lines showed that the combined endogenous and ectopic levels were in the same range in all three lymphoma subtypes and thus an unlikely explanation for the lack of phenotype upon overexpression of miR-150 in HL and DLBCL.

As a second possible explanation we determined whether miR-150 targets MYB and ZDHHC11 in HL because these cell lines showed the weakest effect on cell growth upon miR-150 overexpression. Probes for both MYB and ZDHHC11 were among the most enriched targets, with IP enrichment levels similar to those observed in BL [11]. Effective targeting of MYB by miR-150 was also confirmed at the protein level. Together, these data showed that miR-150 efficiently targets both MYB and ZDHHC11 transcripts in HL and that the lack of inducing a phenotype could not be explained by lack of targeting these two transcripts. 

A third possible explanation is that in BL the effect of miR-150 on cell growth is caused by a combined regulation of multiple target genes including MYB and ZDHHC11, but also other genes. This could imply that in HL the efficiency of miR-150 for targeting MYB and ZDHHC11 is insufficient to cause a clear effect on cell growth. It has indeed been shown that the miR-150 target gene repertoire can highly differ between different hematopoietic malignancies, e.g., MYB, FLT3, CBL and EGR2 in MLL-rearranged AML cells [25,26], and DKC1 and AKT2 in NK/T-cell lymphoma [8]. EGR2 is enriched in the IP fraction upon miR-150 OE in ST486, while it is not expressed in DG75 and not enriched in the IP fractions of the two HL cell lines that do not show a phenotype (L428 and SUPHD1) upon miR-150 overexpression. None of the other reported miR-150 target genes were expressed (FLT3) or IP enriched (CBL, DKC1 and AKT2). Analysis of other genes specifically enriched in the AGO2-IP of BL or HL revealed no obvious candidates that might explain the lack of a phenotype in HL. As our AGO2-IP analysis was focused on protein coding genes, it is possible that next to targeting MYB and ZDHHC11 transcripts, specific ncRNAs (e.g., lncRNAs or circRNAs) are targeted by miR-150 in only BL or HL. Therefore, it remains unclear why miR-150 overexpression does not show a clear phenotype in HL.

We were unable to define the miR-150 target genes in DLBCL cell lines, as the lentiviral infection efficiencies were insufficient to allow isolation of sufficient infected cells for the AGO2-IP procedure. We expect that the efficiencies for the targeting of miR-150 to MYB and ZDHHC11 in DLBCL will also be good but this needs to be established. Thus, we cannot rule out that in DLBCL the limited phenotype upon miR-150 overexpression is related to poor efficiency to target MYB and ZDHHC11. A second limitation of our study is that we used a subset of the cell lines for studying the effect of miR-150 overexpression on growth. Therefore, we cannot exclude the possibility that miR-150 may inhibit growth in other HL or DLBCL cell lines. Lastly, as the efficiencies of the shRNAs against MYC, MYB and ZDHHC11 were not retested in all cell lines, we cannot rule out that differences in the strength of the phenotype are caused by differences in shRNA efficiencies between cell lines.

## 5. Conclusions

In conclusion, although MYC, MYB and ZDHHC11 all have important roles in the growth of HL and DLBCL, the network, as defined in BL with a critical role of miR-150, is not broadly applicable to other GC B-cell derived lymphoma subtypes. Based on our data, the induction of miR-150 might present an attractive therapeutic approach for BL, but not for other GC B-cell derived lymphomas. The lymphoma specific role of ZDHHC11 remains to be elucidated and studying the individual ZDHHC11 products might provide further mechanistic insights in different B-cell lymphoma subtypes.

## Data Availability

Array data have been submitted at GEO under number GSE188310.

## Supplementary Materials

The following are available online at www.mdpi.com/article/10.3390/genes13020227/s1, Figure S1: Expression of MYC, MYB, miR-150 and all ZDHHC11 transcripts in B-cell lymphoma cell lines, Figure S2: Original immunoblots for MYC, MYB and GAPDH in HL and DLBCL cell lines, Figure S3: Expression of ZDHHC11 in primary cases of cHL and DLBCL, Figure S4: Knockdown efficiency tests of shRNAs directed against MYC and MYB, Figure S5: Efficiency of miR-150 overexpression in BL, HL and DLBCL cell lines, Figure S6: Efficiency of the AGO2 RNA Immuno Precipitation assessed by Western blot and qRT-PCR, Figure S7: Original immunoblotsshowing MYB levels in HL cell lines upon overexpression of miR-150, Figure S8: Overlap of miR-150 targets identified in HL and BL cell lines, Table S1: Cell lines and culturing media, Table S2: Sequences of the qRT-PCR primers, Table S3: Antibodies used for western blot analysis, Table S4: MiR-150 target genes identified by AGO2-RIP-Chip in L428 and SUPHD1-miR-150 cells.
