# Supplementary material for: The Role of the MYC/miR-150/MYB/ZDHHC11 Network in Hodgkin Lymphoma and Diffuse Large B-Cell Lymphoma
Source: Genes (Basel). 2022 Jan 25;13(2):227. doi: 10.3390/genes13020227 (PMC8871936; doi:10.3390/genes13020227)
Supplement: Supplementary file 1 [file genes-13-00227-s001.zip › supplementary tables.pdf]

Supplementary Table S1. Cell lines and culturing media

	Type	EBV	Origin	Culturing media	FBS
HEK 293T	human embryonic kidney cell	-	ATCC	DMEM	10%
DG75	BL	-	DSMZ	RPMI-1640	10%
CA46	BL	-	DSMZ	RPMI-1640	10%
ST486	BL	-	ATCC	RPMI-1640	20%
BL41	BL	-	DSMZ	RPMI-1640	10%
BL65	BL	+	[1]	RPMI-1640	20%
Namalwa	BL	+	DSMZ	RPMI-1640	10%
Jijoye	BL	+	DSMZ	RPMI-1640	10%
Raji	BL	+	DSMZ	RPMI-1640	10%
Ramos	BL	-	ATCC	RPMI-1640	10%
L428	HL, nodular sclerosis	-	DSMZ	RPMI-1640	10%
L1236	HL, mixed cellularity	-	DSMZ	RPMI-1640	10%
KMH2	HL, mixed cellularity	-	DSMZ	RPMI-1640	10%
DEV	HL, nodular lymphocyte-predominant	-	[2]	RPMI-1640	20%
L540	HL, nodular sclerosis	-	DSMZ	RPMI-1640	20%
L591	HL, nodular sclerosis	+	DSMZ	RPMI-1640	10%
SUPHD1	Hl, nodular sclerosis	-	DSMZ	McCoy 5A	20%
HDLM2	HL, nodular sclerosis	-	DSMZ	RPMI-1640	10%
U2932	DLBCL, ABC	-	DSMZ	RPMI-1640	10%
Ocily3	DLBCL, ABC	-	DSMZ	RPMI-1640	20%
SUDHL16	DLBCL, GCB	-	DSMZ	RPMI-1640	20%
SUDHL10	DLBCL, GCB	-	DSMZ	RPMI-1640	20%
SUDHL6	DLBCL, GCB	-	DSMZ	RPMI-1640	10%
SUDHL5	DLBCL, GCB	-	DSMZ	RPMI-1640	10%
SUDHL4	DLBCL, GCB	-	DSMZ	RPMI-1640	10%
SUDHL2	DLBCL, ABC	-	DSMZ	RPMI-1640	20%
WSU-DLCL2	DLBCL, GCB	-	DSMZ	RPMI-1640	10%

1. Lenoir GM, Vuillaume M, Bonnardel C. The use of lymphomatous and lymphoblastoid cell lines in the study of Burkitt's lymphoma. IARC Sci Publ. 1985;(60):309-18. PMID: 3934070.
2. Atayar C, Kok K, Kluiver J, Bosga A, van den Berg E, van der Vlies P, Blokzijl T, Harms G, Davelaar I, Sikkema-Raddatz B, Martin-Subero JI, Siebert R, Poppema S, van den Berg A. BCL6 alternative breakpoint region break and homozygous deletion of 17q24 in the nodular lymphocyte predominance type of Hodgkin's lymphoma-derived cell line DEV. Hum Pathol. 2006 Jun;37(6):675-83. doi: 10.1016/j.humpath.2006.01.018. PMID: 16733207.

Supplementary Table S2. Sequences of the qRT-PCR primers

Name	Sequence (5'-3')
MYC-F	GCTCATTTCTGAAGAGGACTTGTTG
MYC-R	TTACGCACAACACTTCCGTAGCT
MYB-F	CCAACTGTTCACGCAGACCT
MYB-R	CTTCTGATGCTGGTGCCATT
ZDHHC11_ALL-F	CACTTGGGCTGCAACAAGAA
ZDHHC11_ALL-R	GGTGGGGTTTCAGGGTAGAAG
pcZDHHC11-F	TGTCGAGCACTCCCCAGA
pcZDHHC11-R	GCAACGGAAAACGGCTCTC
lncZDHHC11-F	AGCACTTCCTGAAAGCCAGC
lncZDHHC11-R	GAAGAACGCAACAGGCATCG
circZDHHC11-F	GGCATCCTCTGTATTTTAATGAACTCT
circZDHHC11-R	GTTGCAGCCAGAAGCCAAG
U6-F	TGGAACGATACAGAGAAGATTAGCA
U6-R	AAAATATGGAACGCTTCACGAATT

F = forward primer, R = reverse primer, S = sense sequence, AS = antisense sequence.

Supplementary Table S3. Antibodies used for western blot analysis.

Name	Dilution	Catalog number, company
rabbit monoclonal anti-c-MYC [Y69]	1:1000	ab32072, Abcam, Cambridge, UK
rat monoclonal anti-c-Myb [ANA236B] (C-terminal)	1:500	ab169111, Abcam, Cambridge, UK
rabbit monoclonal anti-c-Myb (phospho S11) [EP769Y] (N-terminal)	1:500	ab45150, Abcam, Cambridge, UK
mouse monoclonal anti-GAPDH antibody	1:50000	NB600-502, Novus Biologicals, UK
mouse monoclonal anti-AGO2 antibody (clone 2E12-1C9)	1:400	H00027161-M01, Abnova, Taiwan
goat anti-Rabbit Immunoglobulins/HRP	1:1000	P0448, Dako, USA
goat anti-mouse Immunoglobulins/HRP	1:1000	P0447, Dako, USA
rabbit anti-goat immunoglobulins/HRP	1:1000	P0449, Dako, USA

Supplementary Table S4. MiR-150 target genes identified by AGO2-RIP-Chip in L428 and SUPHD1-miR-150 cells.

		L428			SUPHD1		
Probe name	Gene symbol	miR-150 IP/TF	EV IP/TF	miR150/EV	miR-150 IP/TF	EV IP/TF	miR150/EV
A_24_P153456	ZDHHC11	26.7	1.0	26.7	5.7	1.0	5.7
A_33_P3344204	ZDHHC11	25.5	1.0	25.5	6.4	1.0	6.4
A_33_P3345643	ZDHHC11B	22.5	1.0	22.5	6.2	1.0	6.2
A_23_P31073	MYB	4.2	1.0	4.2	4.5	1.1	4.1
A_23_P20882	ATP6V1G1	2.2	1.0	2.2	2.5	1.0	2.5
A_23_P80778	DIRC2	4.7	2.3	2.0	10.5	4.9	2.1
A_23_P115467	S100A5	6.0	1.0	6.0	1.0	#N/A	#N/A
A_21_P0001246	LOC101927851	8.8	1.5	5.8	1.0	2.5	0.4
A_21_P0007561	XLOC_009764	14.1	2.7	5.2	#N/A	#N/A	#N/A
A_21_P0000463	SNORD105B	15.5	3.2	4.9	1.0	1.2	0.8
A_21_P0000260	SNORD58A	9.9	2.0	4.9	1.0	1.3	0.8
A_33_P3243405	GPR182	16.0	3.4	4.8	#N/A	#N/A	#N/A
A_33_P3419945	Lnc-PTPN9-1	9.1	2.1	4.3	1.7	2.3	0.7
A_21_P0000361	SNORA79	7.7	1.8	4.2	#N/A	#N/A	#N/A
A_23_P136870	MAGEA6	4.2	1.0	4.2	#N/A	#N/A	#N/A
A_33_P3249529	PCNX	8.6	2.0	4.2	1.0	1.0	1.0
A_21_P0000354	SCARNA8	8.8	2.1	4.2	5.0	9.2	0.5
A_21_P0011028	LOC102724910	11.0	2.7	4.1	1.7	2.6	0.6
A_33_P7314857	LOC100506085	4.0	1.0	4.0	#N/A	#N/A	#N/A
A_33_P3278220	RABEPK	10.3	2.6	4.0	1.2	2.2	0.6
A_33_P3367396	FAM177B	4.0	1.0	4.0	#N/A	#N/A	#N/A
A_21_P0008515	XLOC_010856	15.3	4.0	3.9	1.0	1.0	1.0
A_22_P00025249	lnc-CEACAM18-2	3.8	1.0	3.8	1.0	1.0	1.0
A_21_P0000494	SNORA16B	29.6	7.8	3.8	1.5	5.2	0.3
A_19_P00315716	SNORA71A	10.1	2.6	3.8	1.3	2.1	0.6
A_22_P00006901	SCARNA10	5.4	1.4	3.8	1.2	1.8	0.7
A_22_P00014382	lnc-SEZ6L2-1	6.3	1.7	3.8	1.1	1.9	0.5
A_22_P00025115	LINC02332	4.0	1.1	3.7	1.0	1.0	1.0
A_33_P3417459	SCARNA9L	3.7	1.0	3.7	#N/A	#N/A	#N/A
A_21_P0014782	LOC105376108	3.7	1.0	3.7	1.0	1.0	1.0

A_22_P0002214 1	lnc-TLCD2-2	3.7	1.0	3.6	1.0	#N/A	#N/A
A_22_P0000156 1	lncRNA	4.4	1.2	3.6	1.9	2.2	0.8
A_21_P0000356	SCARNA11	19.0	5.2	3.6	6.3	10.8	0.6
A_33_P3267612	MIR143HG	4.5	1.2	3.6	1.0	1.0	1.0
A_21_P0004252	lnc-SNX18-1	10.0	2.8	3.6	#N/A	#N/A	#N/A
A_33_P3288871	SLC38A1	3.6	1.0	3.6	#N/A	#N/A	#N/A
A_33_P3366493	TPD52L2 pseudogene	4.0	1.1	3.6	#N/A	#N/A	#N/A
A_21_P0005184	lnc-WDR27-1	3.6	1.0	3.6	1.0	1.0	1.0
A_22_P0002098 0	lnc-C7orf45-1	3.6	1.0	3.6	1.0	#N/A	#N/A
A_22_P0001803 6	lnc-ZNF236-1	4.1	1.2	3.5	1.0	1.0	1.0
A_33_P3236416	GPR179	10.3	2.9	3.5	1.0	1.0	1.0
A_21_P0008478	lnc-SERPINA12-1	3.8	1.1	3.5	#N/A	#N/A	#N/A
A_21_P0000222	SNORD46	6.0	1.7	3.5	#N/A	#N/A	#N/A
A_21_P0000563	SNORA80B	15.2	4.4	3.5	3.5	7.0	0.5
A_33_P3331426	XLOC_l2_008130	9.7	2.8	3.5	1.3	2.0	0.7
A_21_P0010155	LOC284825	6.8	2.0	3.4	1.8	3.0	0.6
A_21_P0011549	XLOC_l2_005871	3.4	1.0	3.4	1.0	1.1	0.9
A_33_P3215557	ADAMTS7	3.4	1.0	3.4	1.0	1.0	1.0
A_33_P3219459	TMEM240	3.4	1.0	3.4	1.0	#N/A	#N/A
A_21_P0000310	SNORA12	18.7	5.5	3.4	3.5	6.7	0.5
A_33_P3291776	NANS	3.4	1.0	3.4	1.9	1.0	1.9
A_21_P0007229	lncRNA	9.2	2.7	3.4	1.0	1.0	1.0
A_21_P0000386	SNORD89	5.6	1.7	3.4	#N/A	#N/A	#N/A
A_33_P3346680	CDC37P2	3.4	1.0	3.4	1.0	1.0	1.0
A_22_P0001031 9	lnc-MUC5B-1	3.3	1.0	3.3	#N/A	#N/A	#N/A
A_23_P140967	MEFV	3.3	1.0	3.3	1.0	#N/A	#N/A
A_21_P0000351	SCARNA22	10.2	3.0	3.3	1.8	3.4	0.5
A_21_P0000359	SNORA71D	5.7	1.7	3.3	1.0	1.2	0.8
A_21_P0000317	SNORA22	23.4	7.1	3.3	3.7	11.0	0.3
A_21_P0000350	SCARNA20	5.2	1.6	3.3	1.5	1.8	0.9
A_21_P0000262	SNORA27	18.6	5.7	3.3	8.1	22.9	0.4
A_22_P0001268 7	LOC100507156	3.3	1.0	3.3	1.0	1.0	1.0
A_33_P3628409	PKI55	3.3	1.0	3.3	1.0	#N/A	#N/A
A_23_P129413	DPEP3	3.3	1.0	3.3	1.0	1.0	1.0
A_21_P0006193	lnc-STX17-1	3.2	1.0	3.2	#N/A	#N/A	#N/A
A_21_P0007890	lncRNA	3.3	1.0	3.2	1.0	1.0	1.0

A_33_P3299140	GTF3C5	3.2	1.0	3.2	#N/A	#N/A	#N/A
A_23_P44956	RPL35A	4.6	1.4	3.2	1.9	1.2	1.6
A_21_P0000353	SCARNA23	13.2	4.1	3.2	1.6	#N/A	#N/A
A_21_P0000477	SNORA11B	17.2	5.4	3.2	4.2	7.3	0.6
A_24_P504621	HIGD1AP11	3.2	1.0	3.2	#N/A	#N/A	#N/A
A_33_P3265866	PYY3	3.5	1.1	3.2	#N/A	#N/A	#N/A
A_24_P233078	PYY2	4.0	1.3	3.1	1.0	1.0	1.0
A_33_P3337627	TRPC6	5.9	1.9	3.1	1.5	2.4	0.6
A_22_P0001174 3	lnc-PER2-1	3.1	1.0	3.1	1.0	1.0	1.0
A_33_P3231923	LOC101927285	6.9	2.2	3.1	1.4	1.8	0.8
A_21_P0000322	SNORA34	39.2	12.6	3.1	8.8	23.6	0.4
A_21_P0000478	SNORA11C	15.2	4.9	3.1	6.2	6.7	0.9
A_24_P21770	YPEL4	3.4	1.1	3.1	1.0	1.0	1.0
A_22_P0001164 3	lnc-PDCD11-2	3.1	1.0	3.1	1.0	1.0	1.0
A_23_P27133	KRT15	3.1	1.0	3.1	1.0	1.0	1.0
A_23_P49674	ARHGEF15	6.1	2.0	3.1	1.0	1.6	0.6
A_23_P50815	TTYH1	3.1	1.0	3.1	1.0	1.0	1.0
A_22_P0000184 7	THOC7-AS1	7.3	2.4	3.1	1.4	1.9	0.7
A_23_P416314	HRASLS5	5.0	1.6	3.1	1.3	1.7	0.7
A_21_P0013455	ZNF767P	8.6	2.8	3.1	1.0	#N/A	#N/A
A_33_P3271196	AMOTL1	3.3	1.1	3.1	1.0	1.0	1.0
A_19_P0031596 7	MLYCD	3.2	1.0	3.1	#N/A	#N/A	#N/A
A_33_P3302428	TNRC6C	5.2	1.7	3.0	1.2	1.8	0.7
A_21_P0000867	ZBTB20-AS1	4.5	1.5	3.0	1.2	1.8	0.7
A_33_P3415491	WDR90	3.0	1.0	3.0	1.0	1.0	1.0
A_33_P3363082	SCARNA5	7.3	2.4	3.0	4.2	7.5	0.6
A_33_P3849275	FHL1	3.0	1.0	3.0	1.0	1.0	1.0
A_22_P0001895 0	TMEM231	3.0	1.0	3.0	1.0	1.0	1.0
A_22_P0000767 3	lnc-HIATL1-1	3.4	1.1	3.0	1.0	1.0	1.0
A_33_P3327140	lncRNA	8.0	2.7	3.0	1.5	1.9	0.8
A_33_P3214803	PRDM13	3.0	1.0	3.0	1.0	1.0	1.0
A_33_P3709317	SNORA28	15.1	5.1	3.0	26.7	35.0	0.8
A_21_P0011923	WDPCP	3.0	1.0	3.0	1.0	1.0	1.0
A_19_P0080243 3	LINC01564	11.0	3.7	3.0	1.0	1.0	1.0
A_21_P0005807	lnc-GFRA2-1	8.9	3.0	3.0	1.0	#N/A	#N/A

A_19_P00808834	LINC01478	3.0	1.0	3.0	#N/A	#N/A	#N/A
A_21_P0011822	lnc-ZAP70-2	3.2	1.1	2.9	#N/A	#N/A	#N/A
A_23_P50146	SIGLEC15	3.4	1.1	2.9	1.0	1.0	1.0
A_33_P3334180	PLCH2	3.3	1.1	2.9	1.0	1.0	1.0
A_19_P00801735	SEPT7-AS1	3.1	1.1	2.9	1.0	1.0	1.0
A_24_P152468	LOC100128364	3.0	1.0	2.9	1.0	1.0	1.0
A_22_P00006567	lnc-FLYWCH2-1	3.0	1.0	2.9	#N/A	#N/A	#N/A
A_21_P0000340	SNORA60	4.2	1.4	2.9	1.1	2.2	0.5
A_23_P396981	CCDC66	4.0	1.4	2.9	1.0	#N/A	#N/A
A_33_P3351606	MIR124-2HG	2.9	1.0	2.9	1.0	1.0	1.0
A_33_P3271975	LOC100132368	2.9	1.0	2.9	1.0	#N/A	#N/A
A_23_P68910	SSTR3	3.6	1.2	2.9	1.0	1.0	1.0
A_24_P924862	RAPH1	3.6	1.3	2.9	1.5	1.4	1.1
A_21_P0000237	SNORA10	6.2	2.2	2.9	2.1	3.5	0.6
A_21_P0009268	lnc-MAP2K6-2	11.5	4.0	2.9	1.0	1.0	1.0
A_22_P00017446	lnc-VPS4A-1	3.9	1.4	2.9	1.0	1.0	1.0
A_33_P3236340	LOC100133131	4.2	1.5	2.9	1.6	2.3	0.7
A_22_P00003471	lnc-CCDC69-1	2.9	1.0	2.9	#N/A	#N/A	#N/A
A_21_P0000336	SNORA54	31.2	11.0	2.8	22.8	30.0	0.8
A_33_P3311046	OR2M7	2.8	1.0	2.8	#N/A	#N/A	#N/A
A_22_P00009316	LOC100507205	3.3	1.2	2.8	1.0	1.4	0.7
A_21_P0000324	SNORA36A	7.7	2.7	2.8	#N/A	#N/A	#N/A
A_33_P3403643	LOC101059906	2.9	1.0	2.8	1.0	1.0	1.0
A_33_P3322192	RP11-74K11.1	2.8	1.0	2.8	1.0	1.0	1.0
A_33_P3882624	POT1	3.1	1.1	2.8	1.0	1.0	1.0
A_24_P47681	CAND1	4.1	1.5	2.8	1.0	1.3	0.8
A_21_P0012967	CTC-338M12.9	3.6	1.3	2.8	1.0	1.0	1.0
A_22_P00016235	SNORD57	7.5	2.7	2.8	1.0	1.0	1.0
A_21_P0000212	SNORA68	5.9	2.1	2.8	1.8	4.0	0.4
A_21_P0007854	lnc-HNF1A-1	12.3	4.4	2.8	1.0	1.0	1.0
A_21_P0003889	FLJ36777	3.0	1.1	2.8	#N/A	#N/A	#N/A
A_23_P42288	VWA7	3.1	1.1	2.8	1.0	1.0	1.0
A_33_P3391375	LANCL3	6.4	2.3	2.8	1.6	2.0	0.8
A_19_P00321789	RP3-523C21.2	2.8	1.0	2.8	1.0	1.0	1.0

A_22_P00005191	lnc-DKK3-1	2.9	1.0	2.7	1.0	1.0	1.0
A_32_P207243	LOC100134253	26.1	9.5	2.7	3.7	9.6	0.4
A_21_P0000493	SCARNA14	8.8	3.2	2.7	4.2	6.7	0.6
A_33_P3335576	LOC100287006	2.7	1.0	2.7	1.0	#N/A	#N/A
A_33_P3274245	ENDOV	3.5	1.3	2.7	1.0	#N/A	#N/A
A_33_P3368109	EIF4BP3	2.7	1.0	2.7	1.0	1.0	1.0
A_21_P0000308	SNORA9	8.3	3.1	2.7	4.3	9.5	0.5
A_23_P119448	PPP6R1	10.7	3.9	2.7	1.0	1.0	1.0
A_19_P00805812	lnc-UQCRFS1-9	11.1	4.1	2.7	#N/A	#N/A	#N/A
A_33_P3386686	LOC100132874	2.7	1.0	2.7	1.0	1.0	1.0
A_21_P0009285	lnc-C1QTNF1-1	2.7	1.0	2.7	1.0	#N/A	#N/A
A_33_P3312504	PSD4	2.7	1.0	2.7	1.0	1.0	1.0
A_21_P0011573	AC004448.5	2.7	1.0	2.7	1.0	1.0	1.0
A_23_P115573	SHISA4	2.7	1.0	2.7	1.0	1.0	1.0
A_22_P00003107	lnc-MFAP4-3	2.7	1.0	2.7	1.0	1.0	1.0
A_33_P3356577	SIRPB1	2.7	1.0	2.7	1.0	1.0	1.0
A_33_P3629247	ANKMY1	3.2	1.2	2.7	1.0	1.2	0.9
A_22_P00002486	lnc-C16orf13-3	3.0	1.1	2.7	1.0	1.0	1.0
A_24_P307289	TMEM95	3.5	1.3	2.7	1.0	1.0	1.0
A_21_P0000325	SNORA37	15.7	5.9	2.7	5.0	9.3	0.5
A_21_P0000369	SNORA16A	19.5	7.3	2.7	1.4	3.8	0.4
A_22_P00007888	LINC00092	3.1	1.2	2.7	1.0	1.0	1.0
A_24_P292470	UCP3	2.7	1.0	2.7	1.0	1.0	1.0
A_22_P00011325	lnc-OST4-2	2.7	1.0	2.7	1.0	1.0	1.0
A_33_P3452003	LOC143286	3.6	1.4	2.7	1.8	1.9	0.9
A_33_P3255434	MEG3	3.1	1.2	2.6	#N/A	#N/A	#N/A
A_22_P00008937	C12orf80	2.6	1.0	2.6	1.0	1.0	1.0
A_21_P0004128	lnc-EMB-3	2.6	1.0	2.6	1.0	#N/A	#N/A
A_33_P3241250	LOC100132217	2.8	1.1	2.6	1.0	1.0	1.0
A_23_P15357	LGALS3BP	2.7	1.0	2.6	1.0	1.0	1.0
A_33_P3376478	CYP17A1	2.7	1.0	2.6	#N/A	#N/A	#N/A
A_24_P51683	CDK5R2	2.6	1.0	2.6	1.0	1.0	1.0
A_24_P106542	RSPO3	3.6	1.4	2.6	1.0	1.1	0.9
A_21_P0006079	LINC01506	2.9	1.1	2.6	1.0	#N/A	#N/A

A_22_P0000295 0	lnc-C5orf47-2	2.6	1.0	2.6	1.0	1.0	1.0
A_33_P3228739	LRRC3C	2.6	1.0	2.6	1.0	1.0	1.0
A_23_P17307	MRGBP	2.6	1.0	2.6	2.3	2.2	1.1
A_33_P3213419	LOC100129447	2.6	1.0	2.6	1.0	1.0	1.0
A_22_P0001049 3	lnc-NBAS-1	4.3	1.7	2.6	1.0	1.0	1.0
A_22_P0001212 8	lnc-POLR1E-2	2.6	1.0	2.6	1.0	1.0	1.0
A_23_P67896	SCN3A	3.0	1.2	2.6	#N/A	#N/A	#N/A
A_22_P0000127 0	lnc-ANKRD53-1	2.9	1.1	2.6	1.0	1.0	1.0
A_33_P3310976	lnc-CDH4-1	2.6	1.0	2.6	1.0	1.0	1.0
A_21_P0013089	XLOC_l2_013056	2.6	1.0	2.6	1.0	1.0	1.0
A_22_P0000414 2	lnc-CLDN6-2	3.5	1.4	2.6	1.0	1.0	1.0
A_21_P0000295	SNORA67	11.8	4.6	2.6	1.8	3.3	0.5
A_33_P3287379	COG8	3.3	1.3	2.6	1.0	1.0	1.0
A_21_P0011562	XLOC_l2_005933	3.2	1.3	2.6	1.0	1.0	1.0
A_21_P0012148	XLOC_l2_008632	2.5	1.0	2.5	1.0	1.3	0.8
A_19_P0031576 4	LOC644277	2.7	1.1	2.5	1.0	1.0	1.0
A_32_P52330	LOC113230	2.8	1.1	2.5	1.0	1.0	1.0
A_21_P0000331	SNORA44	17.7	7.0	2.5	#N/A	#N/A	#N/A
A_33_P3410821	LOC100134360	3.8	1.5	2.5	1.0	1.0	1.0
A_21_P0010382	lnc-LARGE1-11	2.5	1.0	2.5	#N/A	#N/A	#N/A
A_33_P3383696	SPEG	6.9	2.7	2.5	13.8	12.4	1.1
A_19_P0031584 3	SCARNA16	5.7	2.2	2.5	4.1	6.4	0.6
A_21_P0000347	SNORA76C	3.0	1.2	2.5	1.0	1.0	1.0
A_21_P0003242	lnc-EPHA6-1	2.7	1.1	2.5	1.0	1.0	1.0
A_22_P0001988 6	HNF4A-AS1	2.5	1.0	2.5	1.0	1.0	1.0
A_21_P0009382	LINC01563	2.5	1.0	2.5	1.0	1.0	1.0
A_21_P0000126	TGFBR3L	2.8	1.1	2.5	1.0	1.0	1.0
A_33_P3247624	REP15	11.6	4.6	2.5	#N/A	#N/A	#N/A
A_21_P0000300	SNORA48	7.4	2.9	2.5	1.0	1.6	0.6
A_24_P255384	RPL31P43	2.5	1.0	2.5	1.0	1.2	0.8
A_21_P0008580	LOC101929151	2.5	1.0	2.5	1.0	1.0	1.0
A_22_P0002391 9	lnc-MEGF10-1	2.9	1.2	2.5	1.0	1.0	1.0
A_21_P0007948	lnc-AL359392.1-2	2.9	1.2	2.5	1.2	2.2	0.5

A_21_P0000225	SNORD83B	7.7	3.1	2.5	1.0	1.0	1.0
A_23_P352535	PPP1R16B	2.5	1.0	2.5	1.0	1.0	1.0
A_22_P00006862	TCEB3-AS1	3.0	1.2	2.5	1.0	1.0	1.0
A_21_P0001708	lnc-HMCN1-2	11.4	4.6	2.5	1.0	1.0	1.0
A_23_P75790	MYRF	2.6	1.0	2.5	1.0	1.0	1.0
A_21_P0008072	lnc-AL445989.1-2	2.7	1.1	2.5	1.0	1.0	1.0
A_22_P00016483	LOC148413	2.5	1.0	2.5	1.0	2.0	0.5
A_32_P207124	CT47A11	2.8	1.1	2.5	1.0	1.0	1.0
A_33_P3258324	LOC102724279	2.7	1.1	2.5	1.0	1.0	1.0
A_33_P3358233	NES	3.0	1.2	2.5	1.0	1.0	1.0
A_33_P3306207	KLRG2	2.8	1.1	2.5	1.0	1.0	1.0
A_33_P3279124	FAM21C	2.5	1.0	2.5	1.0	1.0	1.0
A_19_P00317653	LINC00969	2.5	1.0	2.5	1.0	1.0	1.0
A_22_P00000643	lnc-ACSM5-1	2.5	1.0	2.5	#N/A	#N/A	#N/A
A_22_P00000427	BOLA3-AS1	2.9	1.2	2.5	1.0	1.0	1.0
A_19_P00325604	LINC-ROR	2.5	1.0	2.5	#N/A	#N/A	#N/A
A_33_P3350673	HOPX	3.2	1.3	2.5	#N/A	#N/A	#N/A
A_23_P48455	AMN	2.5	1.0	2.5	1.0	1.0	1.0
A_21_P0006850	lnc-LYZL1-2	2.5	1.0	2.5	1.0	#N/A	#N/A
A_23_P67952	MYCNOS	2.5	1.0	2.5	1.0	1.0	1.0
A_22_P00023006	lnc-CHRNA6-1	2.5	1.0	2.5	1.0	1.0	1.0
A_33_P3311717	TGIF1	2.4	1.0	2.4	1.0	1.0	1.0
A_33_P3258316	XLOC_l2_013837	2.4	1.0	2.4	1.0	1.0	1.0
A_22_P00003321	lnc-CARHSP1-1	2.4	1.0	2.4	1.0	1.0	1.0
A_33_P3287310	TMEM273	2.4	1.0	2.4	1.0	1.0	1.0
A_22_P00017056	lnc-TUBB-7	2.4	1.0	2.4	#N/A	#N/A	#N/A
A_22_P00016785	LINC00967	2.9	1.2	2.4	1.0	1.0	1.0
A_22_P00013897	lnc-RPS21-2	3.1	1.3	2.4	1.0	1.0	1.0
A_21_P0009788	lnc-ZNF793-1	2.4	1.0	2.4	1.0	1.0	1.0
A_33_P3380751	ST8SIA1	2.4	1.0	2.4	1.4	2.2	0.6
A_23_P47282	ST14	2.4	1.0	2.4	1.0	#N/A	#N/A

A_21_P0002612	lnc-GPR39-2	2.4	1.0	2.4	1.0	#N/A	#N/A
A_22_P00005332	lnc-DOLPP1-1	2.4	1.0	2.4	1.0	1.0	1.0
A_21_P0000307	SNORA2B	9.3	3.8	2.4	4.3	8.6	0.5
A_33_P3239759	PPAN-P2RY11	3.9	1.6	2.4	1.0	1.0	1.0
A_21_P0013085	XLOC_l2_013031	3.3	1.4	2.4	1.1	1.6	0.7
A_22_P00014771	lnc-SLC36A4-1	3.1	1.3	2.4	1.0	1.0	1.0
A_21_P0002398	lnc-KIDINS220-6	3.2	1.3	2.4	1.0	1.0	1.0
A_21_P0012616	XLOC_l2_010854	2.6	1.1	2.4	1.0	1.0	1.0
A_23_P254212	RPA4	7.0	2.9	2.4	1.0	1.0	1.0
A_33_P3239102	GOLGA6L10	4.4	1.8	2.4	1.2	1.7	0.7
A_33_P3350202	MOCS3	2.4	1.0	2.4	1.0	1.0	1.0
A_33_P3323019	LOC729856	2.6	1.1	2.4	1.0	1.0	1.0
A_33_P3344991	TBC1D3L	2.7	1.1	2.4	1.0	1.0	1.0
A_22_P00012807	lnc-RAP1GDS1-3	2.4	1.0	2.4	1.0	#N/A	#N/A
A_23_P373031	CACNA1C	2.4	1.0	2.4	1.0	1.0	1.0
A_22_P00014270	MFI2-AS1	3.6	1.5	2.4	1.0	1.0	1.0
A_33_P3329462	DLEU1-AS1	3.1	1.3	2.4	1.0	#N/A	#N/A
A_33_P3284715	SCARNA7	4.4	1.9	2.4	1.0	1.0	1.0
A_21_P0004750	lnc-SUPT3H-1	9.5	4.0	2.4	1.0	1.0	1.0
A_23_P89155	CDK3	3.9	1.6	2.4	1.0	1.7	0.6
A_33_P3285868	CYGB	2.4	1.0	2.4	1.0	1.0	1.0
A_22_P00011520	lnc-PAXIP1-1	2.9	1.2	2.4	1.0	1.0	1.0
A_33_P3247403	TOR3A	2.4	1.0	2.4	1.0	1.0	1.0
A_33_P3221960	IL18RAP	3.0	1.3	2.4	1.0	2.0	0.5
A_33_P3293913	BICC1	8.2	3.5	2.4	#N/A	#N/A	#N/A
A_22_P00024468	lnc-CTNNA2-1	3.2	1.3	2.4	1.0	1.0	1.0
A_33_P3335371	MAML3	2.4	1.0	2.4	1.0	1.0	1.0
A_24_P368943	EVX1	2.4	1.0	2.4	1.0	1.0	1.0
A_21_P0000299	SNORA19	24.9	10.5	2.4	11.3	17.7	0.6
A_21_P0003216	LOC101927440	4.1	1.7	2.4	1.0	#N/A	#N/A
A_21_P0000236	SNORA64	8.8	3.7	2.4	2.8	3.8	0.7
A_33_P3308153	MTM1	2.4	1.0	2.4	1.0	1.0	1.0
A_33_P3267482	KIAA1804	3.2	1.4	2.4	1.0	1.0	1.0
A_24_P280497	FBRSL1	2.4	1.0	2.4	1.0	1.0	1.0
A_33_P3343981	AATK	3.1	1.3	2.3	1.0	1.0	1.0
A_33_P3401284	RMRP	4.1	1.8	2.3	1.0	1.0	1.0

A_21_P0000479	SNORA11D	14.7	6.3	2.3	4.5	7.2	0.6
A_33_P3381292	lnc-FGF3-3	2.3	1.0	2.3	1.0	1.0	1.0
A_24_P18802	VPS18	7.4	3.1	2.3	1.0	1.0	1.0
A_21_P0004883	lnc-UBR2-1	2.3	1.0	2.3	1.0	1.0	1.0
A_21_P0000218	SNORD35A	3.5	1.5	2.3	#N/A	#N/A	#N/A
A_21_P0000309	SNORA11	5.2	2.2	2.3	2.9	4.6	0.6
A_22_P0000627 8	lnc-FAM76A-1	3.0	1.3	2.3	1.0	1.0	1.0
A_33_P3406828	MAFIP	2.4	1.0	2.3	1.0	1.0	1.0
A_23_P157465	UBXN8	2.6	1.1	2.3	2.0	1.3	1.5
A_33_P3275722	LY6G6D	2.4	1.0	2.3	1.0	1.0	1.0
A_22_P0001489 9	lnc-SLC9A1-1	2.3	1.0	2.3	1.0	1.0	1.0
A_21_P0011595	XLOC_l2_006101	5.7	2.4	2.3	1.0	#N/A	#N/A
A_33_P3318946	HAPLN2	2.9	1.3	2.3	1.0	1.0	1.0
A_21_P0000258	SNORD26	7.3	3.1	2.3	1.0	1.0	1.0
A_22_P0002041 4	lnc-TTC7B-2	2.7	1.2	2.3	1.0	#N/A	#N/A
A_33_P3287113	UVSSA	2.3	1.0	2.3	1.0	1.0	1.0
A_22_P0000041 9	TM4SF19-AS1	2.3	1.0	2.3	1.0	1.0	1.0
A_33_P3365666	DTX2	2.5	1.1	2.3	1.0	1.0	1.0
A_33_P3278826	LTK	3.4	1.5	2.3	1.0	1.0	1.0
A_21_P0000593	SNORA70D	2.9	1.3	2.3	#N/A	#N/A	#N/A
A_22_P0001776 2	CCNT2-AS1	9.7	4.2	2.3	1.0	1.0	1.0
A_22_P0001677 5	TRIM52-AS1	2.6	1.1	2.3	1.0	1.0	1.0
A_21_P0014853	GS1-24F4.2	2.3	1.0	2.3	1.0	1.0	1.0
A_21_P0000348	SNORA80A	4.7	2.0	2.3	1.0	1.0	1.0
A_33_P3239143	ZNF497	2.3	1.0	2.3	1.0	1.0	1.0
A_22_P0002461 9	lnc-RP11-503N18.3.1-3	2.4	1.0	2.3	1.0	1.0	1.0
A_33_P3234540	ABCA17P	2.3	1.0	2.3	1.0	1.0	1.0
A_22_P0001257 0	lnc-PTP4A2-1	2.3	1.0	2.3	1.0	1.0	1.0
A_22_P0000629 0	RAD51-AS1	2.9	1.3	2.3	1.0	1.0	1.0
A_33_P3305105	VWA5A	2.4	1.0	2.3	1.0	#N/A	#N/A
A_19_P0080807 2	lnc-RTL1-2	2.4	1.0	2.3	1.0	1.0	1.0
A_33_P3474250	TRIM44	2.3	1.0	2.3	1.0	1.7	0.6

A_21_P0013166	FOXL3	2.3	1.0	2.3	1.0	1.0	1.0
A_33_P3359354	C1orf86	2.5	1.1	2.3	1.0	1.0	1.0
A_33_P3396459	POLR2H	2.3	1.0	2.3	1.0	1.0	1.0
A_22_P0000192 4	lnc-BAI3-3	2.4	1.1	2.3	1.0	1.0	1.0
A_24_P229871	LINC00469	2.3	1.0	2.3	2.8	4.1	0.7
A_21_P0000303	SNORA75	3.1	1.4	2.3	#N/A	#N/A	#N/A
A_21_P0000341	SNORA61	5.3	2.3	2.3	1.0	1.4	0.7
A_21_P0000474	SNORA38B	9.8	4.3	2.3	#N/A	#N/A	#N/A
A_33_P3798739	LOC286382	2.3	1.0	2.3	1.0	1.0	1.0
A_21_P0000342	SNORA5B	7.2	3.2	2.3	3.0	2.1	1.5
A_33_P3412556	PPIAP28	2.3	1.0	2.3	1.0	#N/A	#N/A
A_21_P0007321	lnc-SOX6-1	9.4	4.2	2.3	1.0	1.0	1.0
A_33_P3303519	CLEC12B	3.0	1.3	2.3	1.1	2.3	0.5
A_22_P0001468 5	lnc-SLC25A21-1	2.6	1.1	2.3	1.0	1.0	1.0
A_33_P3338360	SCARNA13	5.2	2.3	2.3	3.1	2.9	1.0
A_23_P325726	ACOT11	2.8	1.2	2.3	1.0	#N/A	#N/A
A_22_P0000759 4	lnc-HDDC3-1	2.3	1.0	2.3	1.0	1.0	1.0
A_33_P3393537	PTAFR	2.4	1.0	2.3	1.0	1.0	1.0
A_19_P0031558 1	LINC01122	4.2	1.9	2.3	1.2	1.5	0.8
A_21_P0004987	lnc-GMDS-4	2.3	1.0	2.3	1.0	1.0	1.0
A_33_P3297468	SLC34A3	2.2	1.0	2.2	1.0	1.0	1.0
A_21_P0009377	lnc-NLGN2-1	2.2	1.0	2.2	1.0	1.0	1.0
A_21_P0002976	lnc-GPR27-2	2.8	1.2	2.2	1.0	1.0	1.0
A_22_P0001006 8	LOC101927204	2.2	1.0	2.2	1.0	1.0	1.0
A_33_P3338152	HIF3A	2.2	1.0	2.2	1.0	1.0	1.0
A_22_P0001592 7	lnc-TCL1B-2	10.0	4.5	2.2	1.0	1.0	1.0
A_22_P0002397 5	PAQR9-AS1	3.2	1.4	2.2	1.0	1.0	1.0
A_33_P3229288	ACE	2.2	1.0	2.2	1.0	1.1	0.9
A_22_P0000376 2	lnc-CDIPT-2	2.2	1.0	2.2	1.0	1.0	1.0
A_33_P3335682	PPP1R14A	2.2	1.0	2.2	1.0	1.0	1.0
A_23_P164341	VAMP2	2.5	1.1	2.2	1.0	1.0	1.0
A_22_P0001122 5	lnc-OPRL1-1	2.4	1.1	2.2	1.0	1.0	1.0

A_22_P0001170 4	lnc-PDZD7-1	2.4	1.1	2.2	1.0	1.0	1.0
A_22_P0001519 5	LOC100996842	2.3	1.0	2.2	1.0	1.0	1.0
A_33_P3308456	PRAC2	2.5	1.1	2.2	1.0	1.0	1.0
A_19_P0031937 2	LINC00969	2.7	1.2	2.2	1.0	1.0	1.0
A_33_P3306327	CELF5	3.8	1.7	2.2	1.2	1.8	0.7
A_23_P111311	AKAP12	2.9	1.3	2.2	#N/A	#N/A	#N/A
A_33_P3309365	SLC25A3P1	2.5	1.1	2.2	1.0	1.0	1.0
A_22_P0001975 7	VDAC1P8	2.6	1.2	2.2	1.0	1.1	0.9
A_33_P3280950	A2M-AS1	2.2	1.0	2.2	1.0	1.0	1.0
A_23_P163711	FAM57B	3.0	1.4	2.2	1.0	1.0	1.0
A_33_P3359683	IL16	2.5	1.2	2.2	1.0	1.0	1.0
A_21_P0013931	THC2673826	2.2	1.0	2.2	#N/A	#N/A	#N/A
A_33_P3227217	SNORA81	27.0	12.3	2.2	27.6	44.4	0.6
A_33_P3374365	LOC100129702	2.2	1.0	2.2	1.0	1.0	1.0
A_33_P3365932	WASH1	2.2	1.0	2.2	1.0	1.0	1.0
A_23_P131060	CYP4F8	2.2	1.0	2.2	1.0	1.0	1.0
A_33_P7618758	LINC01541	6.8	3.1	2.2	2.2	1.9	1.2
A_21_P0000592	SNORA70G	3.3	1.5	2.2	1.4	1.6	0.8
A_21_P0005689	LOC101929622	2.4	1.1	2.2	1.0	1.1	0.9
A_33_P3225298	XKR9	2.2	1.0	2.2	1.0	1.0	1.0
A_22_P0000311 0	lnc-C9orf103-1	2.2	1.0	2.2	1.0	1.0	1.0
A_22_P0001811 2	LOC102724231	2.2	1.0	2.2	1.0	1.0	1.0
A_21_P0006395	lnc-PTGES2-1	2.2	1.0	2.2	1.0	1.0	1.0
A_24_P932736	HMBOX1	11.5	5.3	2.2	1.0	1.0	1.0
A_33_P3383226	GP9	2.2	1.0	2.2	#N/A	#N/A	#N/A
A_23_P424734	EXOC3L1	2.4	1.1	2.1	#N/A	#N/A	#N/A
A_19_P0032138 8	LINC00511	2.2	1.0	2.1	1.0	1.0	1.0
A_33_P3312754	LOC102467146	2.1	1.0	2.1	1.0	1.0	1.0
A_33_P3303176	MRGPRG	3.1	1.4	2.1	1.0	1.0	1.0
A_21_P0000220	SNORD33	4.9	2.3	2.1	1.4	2.4	0.6
A_21_P0000397	SCARNA18	2.6	1.2	2.1	2.5	2.7	0.9
A_24_P359191	SLC6A6	2.1	1.0	2.1	1.0	1.0	1.0
A_23_P313031	LOC101930506	2.4	1.1	2.1	1.0	1.0	1.0
A_21_P0012294	GGTLC4P	2.1	1.0	2.1	1.0	1.0	1.0
A_21_P0000394	SNORD110	6.9	3.3	2.1	1.2	#N/A	#N/A

A_22_P00003569	SNHG18	2.4	1.1	2.1	1.0	1.0	1.0
A_23_P155463	LRRC2	9.8	4.6	2.1	1.0	#N/A	#N/A
A_22_P00004304	lnc-CNTLN-2	2.2	1.0	2.1	1.0	1.0	1.0
A_33_P3354771	MEF2BNB	2.3	1.1	2.1	1.0	1.0	1.0
A_23_P219197	RGS3	2.1	1.0	2.1	3.0	3.3	0.9
A_33_P3359308	KLHL29	2.6	1.3	2.1	1.0	1.0	1.0
A_21_P0000257	SNORD27	10.2	4.8	2.1	1.0	1.0	1.0
A_24_P88079	MUC6	2.1	1.0	2.1	1.0	1.0	1.0
A_21_P0010415	lnc-APOL1-1	2.7	1.3	2.1	1.0	1.0	1.0
A_33_P3399468	LOC100133182	2.1	1.0	2.1	1.0	1.0	1.0
A_22_P00002236	KCNMA1-AS1	8.7	4.1	2.1	#N/A	#N/A	#N/A
A_33_P3248272	UBXN8	2.7	1.3	2.1	1.7	1.3	1.3
A_33_P3411080	FSCN2	2.1	1.0	2.1	1.0	1.0	1.0
A_22_P00013961	lnc-RTL1-2	2.3	1.1	2.1	1.0	1.0	1.0
A_21_P0012105	CRYGEP	2.6	1.2	2.1	1.0	1.0	1.0
A_33_P3400429	UBE3A	2.2	1.1	2.1	1.8	1.4	1.3
A_22_P00007112	lnc-GLUD1-3	2.1	1.0	2.1	1.0	1.0	1.0
A_32_P98732	GCM1	2.1	1.0	2.1	#N/A	#N/A	#N/A
A_22_P00002900	CTBP1-AS2	2.1	1.0	2.1	1.0	1.0	1.0
A_24_P396327	TYW3	2.5	1.2	2.1	1.2	1.7	0.7
A_32_P145153	RPL31	2.1	1.0	2.1	1.2	1.0	1.2
A_33_P3300635	PFKFB2	2.1	1.0	2.1	1.0	1.0	1.0
A_22_P00005789	lnc-EPB41-2	2.1	1.0	2.1	1.0	1.0	1.0
A_33_P3306192	KBTBD13	2.1	1.0	2.1	1.0	1.0	1.0
A_22_P00015513	LOC101929294	2.1	1.0	2.1	1.3	1.2	1.1
A_21_P0000591	SNORA70F	2.7	1.3	2.1	#N/A	#N/A	#N/A
A_21_P0014334	LOC101928557	2.4	1.1	2.1	1.0	1.0	1.0
A_22_P00003512	lnc-CCL1-1	2.6	1.3	2.0	1.0	1.0	1.0
A_24_P131589	CD86	8.0	3.9	2.0	1.0	1.0	1.0
A_33_P3301689	ZNF326	2.0	1.0	2.0	1.0	1.0	1.0
A_21_P0007619	LINC02385	2.0	1.0	2.0	1.0	1.0	1.0
A_33_P3292130	XLOC_l2_013383	2.0	1.0	2.0	1.0	1.0	1.0
A_21_P0000363	SNORA25	7.1	3.5	2.0	16.0	19.7	0.8

A_22_P00024993	LOC102724231	2.0	1.0	2.0	1.0	1.0	1.0
A_22_P00009473	PSMG3-AS1	2.0	1.0	2.0	1.0	1.0	1.0
A_21_P0009324	lnc-CCR7-1	2.5	1.2	2.0	1.0	1.0	1.0
A_23_P107801	C19orf44	2.2	1.1	2.0	1.0	1.0	1.0
A_22_P00013941	lnc-RRP8-1	2.3	1.1	2.0	1.0	1.0	1.0
A_23_P389987	TLX2	4.7	2.3	2.0	1.0	1.0	1.0
A_21_P0002061	LOC101927577	2.9	1.4	2.0	1.0	1.0	1.0
A_21_P0000270	SNORA63	4.6	2.3	2.0	1.7	2.8	0.6
A_24_P120934	GADD45G	3.0	1.5	2.0	1.0	1.0	1.0
A_21_P0001923	LINC01911	9.3	4.6	2.0	1.0	1.0	1.0
A_21_P0000301	SNORA5A	7.3	3.6	2.0	1.9	1.8	1.1
A_33_P3241741	SNORA23	3.7	1.8	2.0	3.8	4.0	1.0
A_33_P3393091	DA197111	2.0	1.0	2.0	#N/A	#N/A	#N/A
A_22_P00009574	LOC100506113	2.3	1.2	2.0	1.0	1.0	1.0
A_22_P00000222	LOC100506585	2.0	1.0	2.0	1.0	1.0	1.0
A_33_P3367171	SLC22A8	2.1	1.0	2.0	1.0	1.0	1.0
A_33_P3410849	C8orf58	2.0	1.0	2.0	1.0	1.0	1.0
A_33_P3219811	PTGDS	2.2	1.1	2.0	1.0	1.0	1.0
A_22_P00017815	LINC00638	2.5	1.2	2.0	1.0	1.0	1.0
A_24_P206344	ZNF746	2.0	1.0	2.0	1.0	1.0	1.0
A_21_P0000251	SNORA65	6.2	3.1	2.0	4.2	4.6	0.9
A_21_P0009735	LOC102723811	3.3	1.6	2.0	#N/A	#N/A	#N/A
A_23_P94009	LSM8	2.2	1.1	2.0	3.2	2.1	1.5
A_21_P0007070	lnc-RPP30-2	9.4	4.7	2.0	1.0	1.0	1.0
A_23_P53467	IKBIP	1.1	1.0	1.1	4.0	1.0	4.0
A_24_P370670	ZMYM6NB	1.9	3.2	0.6	3.2	1.0	3.2
A_22_P00016998	DSCR9	4.0	7.4	0.5	6.1	2.1	3.0
A_23_P25121	FKBP11	6.3	10.3	0.6	8.2	2.8	2.9
A_33_P3267296	FKBP11	5.8	10.0	0.6	6.9	2.4	2.9
A_24_P52697	H19	24.1	37.0	0.7	3.0	1.0	2.9
A_23_P41664	LRRC70	#N/A	#N/A	#N/A	2.9	1.0	2.8
A_24_P931443	GPR68	1.0	1.0	1.0	3.4	1.2	2.8
A_24_P166613	EPDR1	1.0	1.3	0.8	2.8	1.0	2.8
A_33_P3352827	SLAMF1	#N/A	#N/A	#N/A	3.9	1.4	2.7
A_23_P28318	NDUFAF7	1.5	1.0	1.5	2.6	1.0	2.6

A_23_P86283	LAPTM5	1.0	1.3	0.8	2.6	1.0	2.6
A_24_P343271	RMND1	1.2	1.0	1.2	2.5	1.0	2.5
A_24_P393565	ZNF396	#N/A	#N/A	#N/A	2.5	1.0	2.5
A_33_P3412160	CLCC1	1.0	1.0	1.0	2.6	1.0	2.5
A_23_P17287	IAH1	1.0	1.0	1.0	3.8	1.5	2.5
A_19_P0031941 3	lnc-NRG1-2	1.3	3.0	0.4	4.7	1.9	2.5
A_23_P136504	SLC25A21	1.0	1.0	1.0	5.2	2.1	2.5
A_23_P114232	PRDX4	3.1	1.8	1.7	8.9	3.6	2.5
A_23_P77415	OSGIN1	1.9	1.3	1.5	3.4	1.4	2.4
A_24_P810290	PPAPDC1A	1.0	1.7	0.6	2.4	1.0	2.4
A_33_P3262789	REEP6	1.0	1.0	1.0	2.5	1.0	2.4
A_33_P3330811	C8orf59	1.0	1.0	1.0	2.5	1.1	2.4
A_23_P68472	DPM1	1.0	1.0	1.0	3.8	1.6	2.4
A_21_P0011758	XLOC_l2_007097	2.0	4.7	0.4	2.8	1.2	2.4
A_23_P98350	BIRC3	1.1	2.3	0.5	2.3	1.0	2.3
A_21_P0006091	XLOC_007776	1.2	1.5	0.8	2.3	1.0	2.3
A_24_P317762	LY6E	1.0	1.2	0.8	2.3	1.0	2.3
A_21_P0002904	LINC01326	1.1	2.4	0.5	2.3	1.0	2.3
A_21_P0007787	LINC00944	1.8	2.0	0.9	3.6	1.6	2.3
A_33_P3295523	RAC3	1.5	1.3	1.1	2.3	1.0	2.3
A_32_P14762	OOEP	1.1	1.6	0.7	3.6	1.6	2.3
A_23_P53856	N4BP2L2	2.2	1.2	1.9	3.1	1.4	2.3
A_21_P0007481	LINC00944	2.5	2.4	1.1	3.8	1.7	2.2
A_24_P389916	LRRC32	4.7	6.2	0.7	3.4	1.5	2.2
A_23_P98252	ARL2	3.7	6.4	0.6	14.6	6.6	2.2
A_22_P0000513 2	LINC00944	1.6	2.1	0.8	3.5	1.6	2.2
A_23_P385217	ARL8B	1.0	1.0	1.0	2.7	1.2	2.2
A_33_P3255229	SETD7	1.0	1.0	1.0	2.2	1.0	2.2
A_23_P251499	PCOLCE	1.0	1.0	1.0	2.2	1.0	2.2
A_24_P16913	ABCC4	1.0	1.3	0.8	7.9	3.7	2.2
A_21_P0010896	LINC00864	1.0	1.0	1.0	4.2	2.0	2.1
A_21_P0012475	XLOC_l2_010489	3.5	7.9	0.4	3.2	1.5	2.1
A_23_P380318	EGR4	1.5	1.3	1.2	2.7	1.3	2.1
A_23_P107693	ZNF586	#N/A	#N/A	#N/A	2.4	1.2	2.1
A_21_P0007789	LINC00944	#N/A	#N/A	#N/A	3.9	1.9	2.1
A_23_P396867	HM13	1.0	1.0	1.0	2.1	1.0	2.1
A_22_P0001861 7	LOC101927027	1.0	1.3	0.8	2.4	1.2	2.0
A_23_P162355	HOXC5	3.6	5.2	0.7	3.6	1.8	2.0
A_33_P3294449	DCAF6	#N/A	#N/A	#N/A	2.6	1.3	2.0

A_23_P131653	PIGF	1.1	1.0	1.1	3.8	1.9	2.0
A_22_P0001685 8	lnc-TRPT1-1	1.7	2.1	0.8	2.5	1.2	2.0
A_33_P3370832	FAM117B	1.0	1.0	1.0	2.1	1.1	2.0
