# Supplementary figures and images for: The Role of the MYC/miR-150/MYB/ZDHHC11 Network in Hodgkin Lymphoma and Diffuse Large B-Cell Lymphoma
Source: Genes (Basel). 2022 Jan 25;13(2):227. doi: 10.3390/genes13020227 (PMC8871936; doi:10.3390/genes13020227)

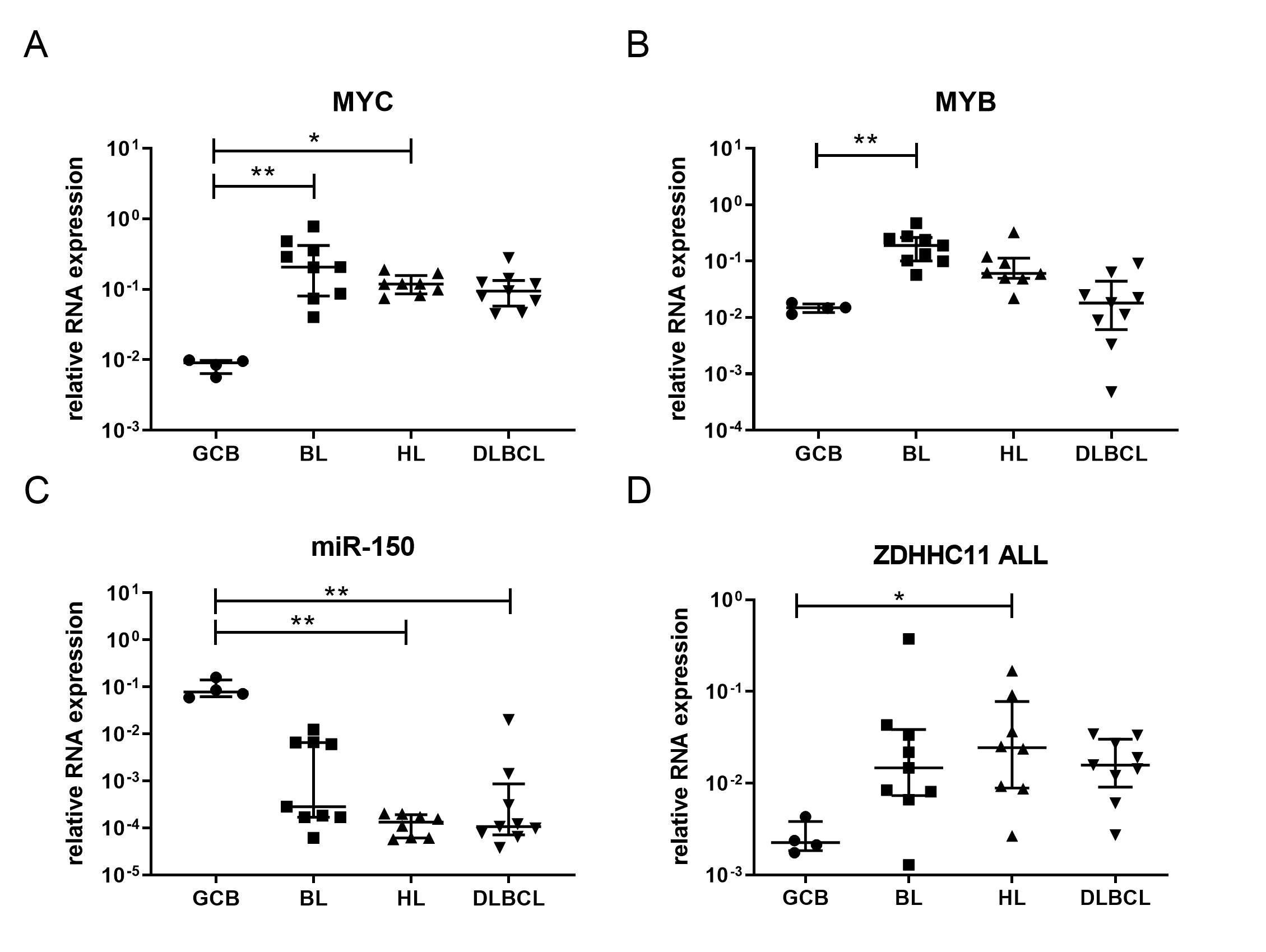

Supplement: Supplementary file 1 [file genes-13-00227-s001.zip › Figure S1.tif]

A

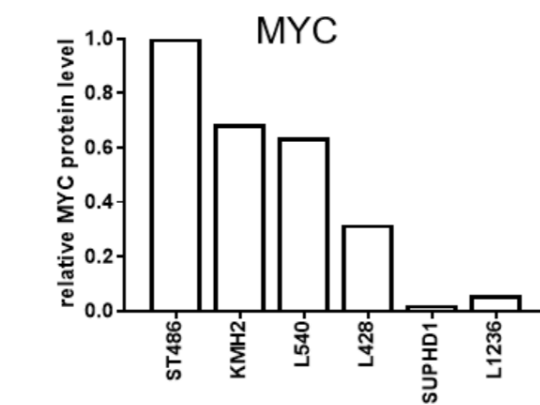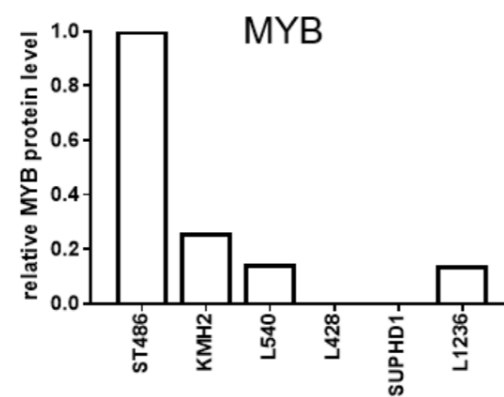

GAPDH

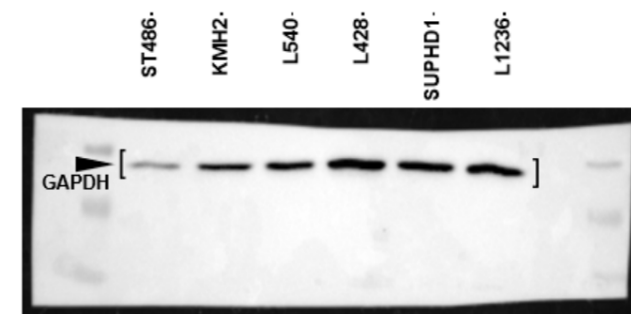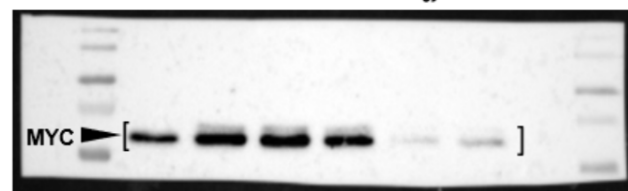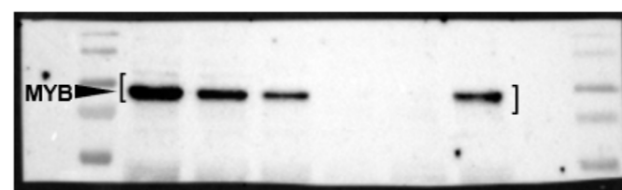

B

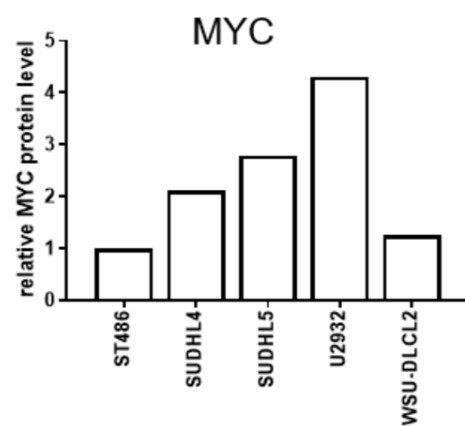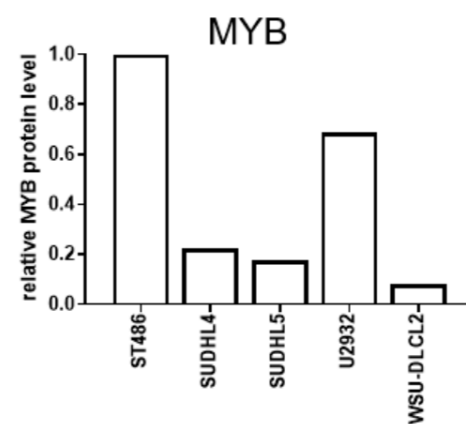

GAPDH

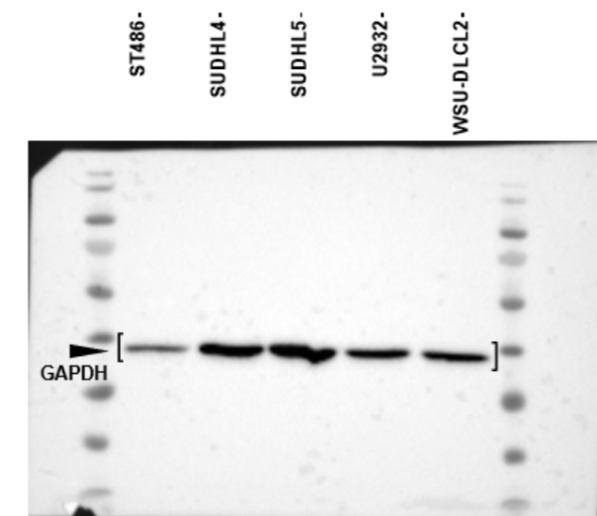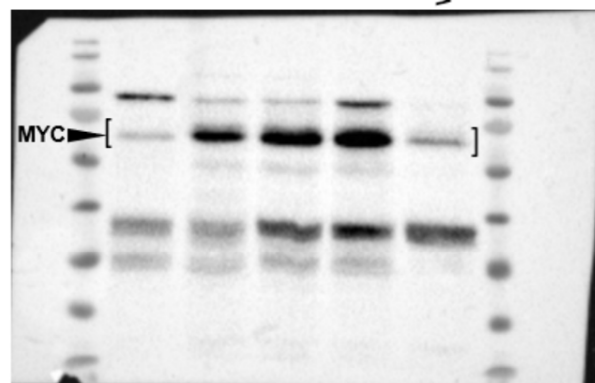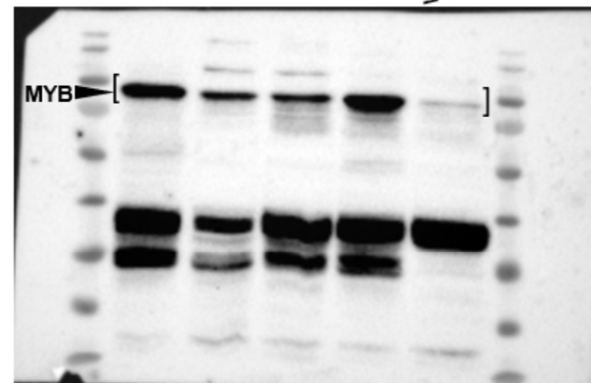

Supplement: Supplementary file 1 [file genes-13-00227-s001.zip › Figure S2.pdf]

A

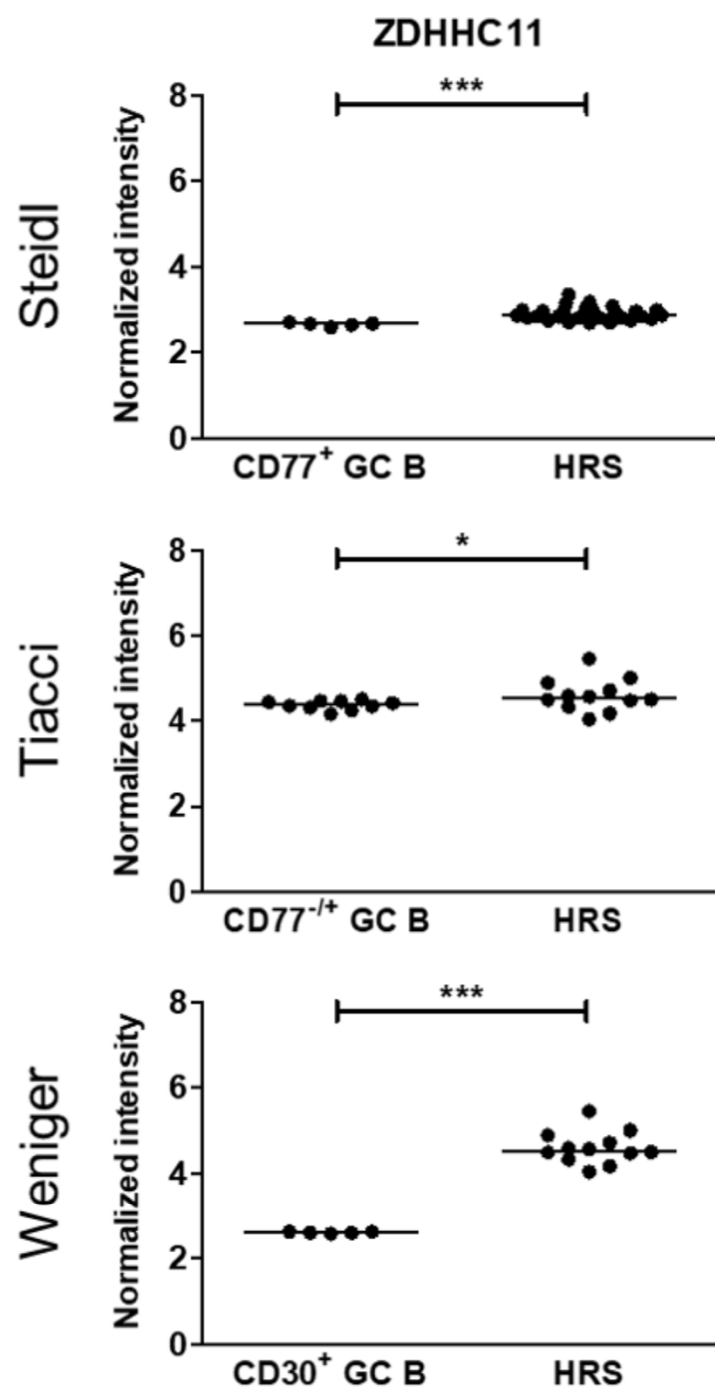

B

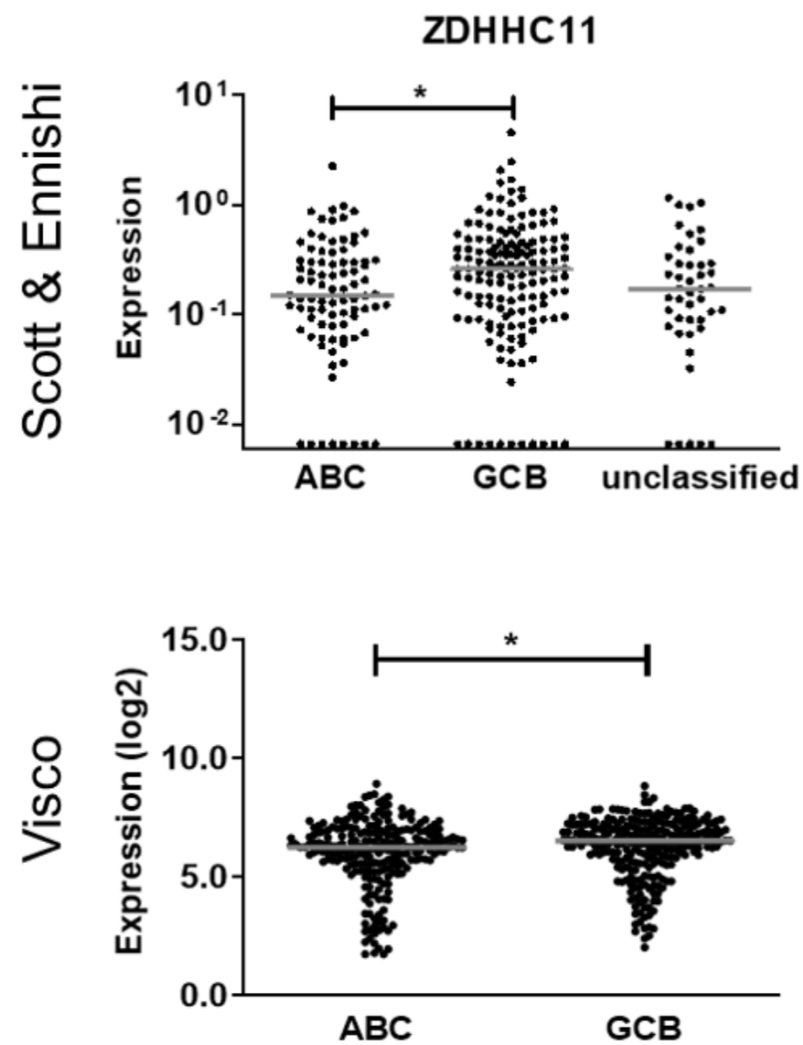

Supplement: Supplementary file 1 [file genes-13-00227-s001.zip › Figure S3.pdf]

A

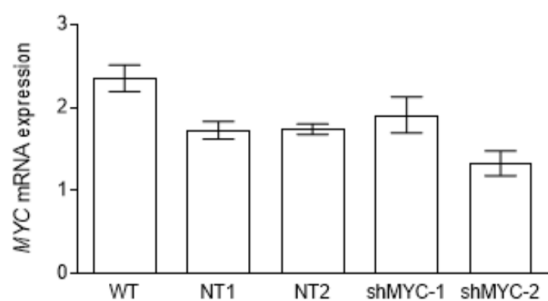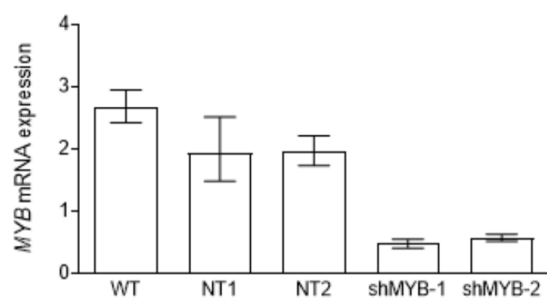

B

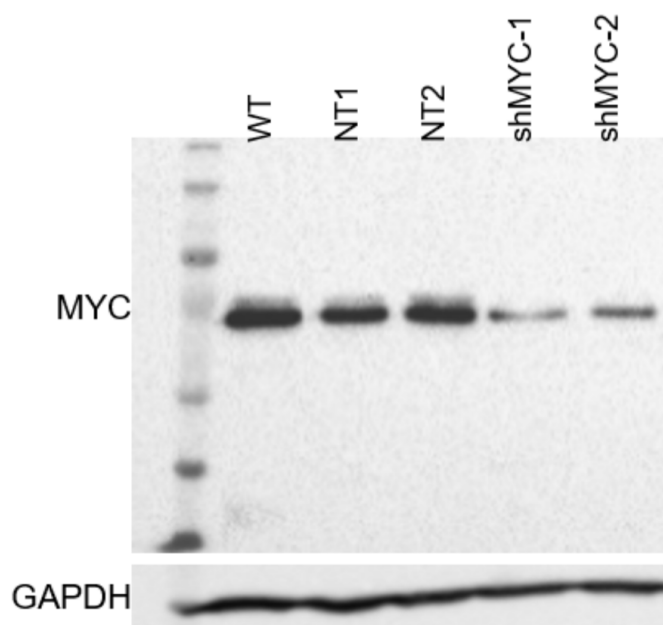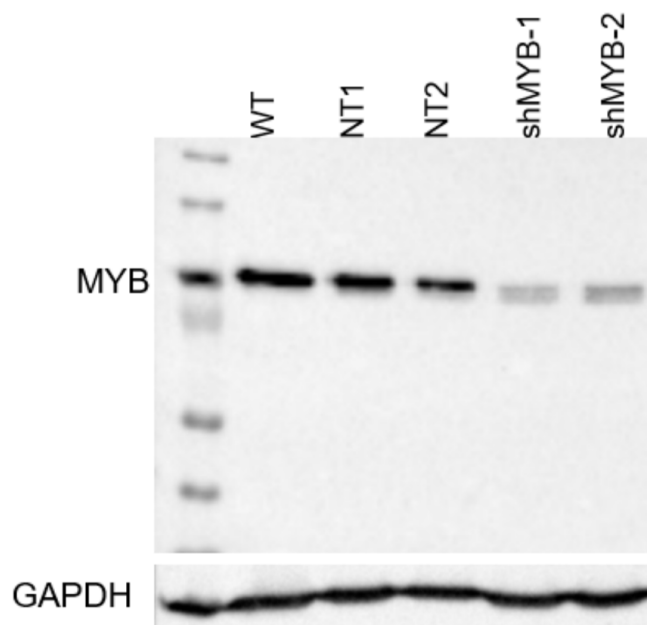

C

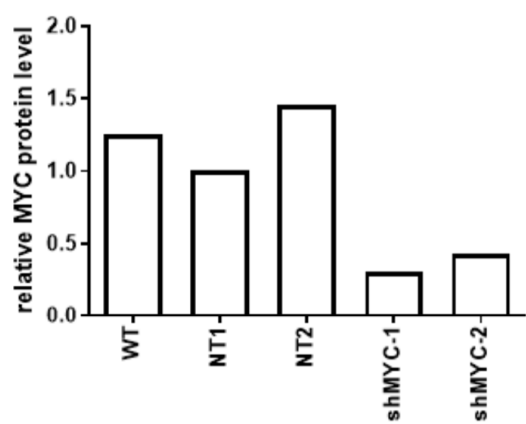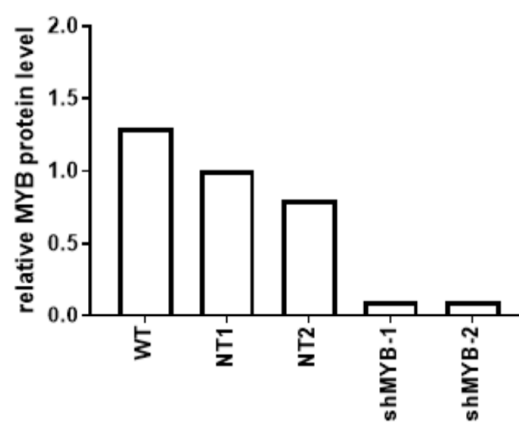

Supplement: Supplementary file 1 [file genes-13-00227-s001.zip › Figure S4.pdf]

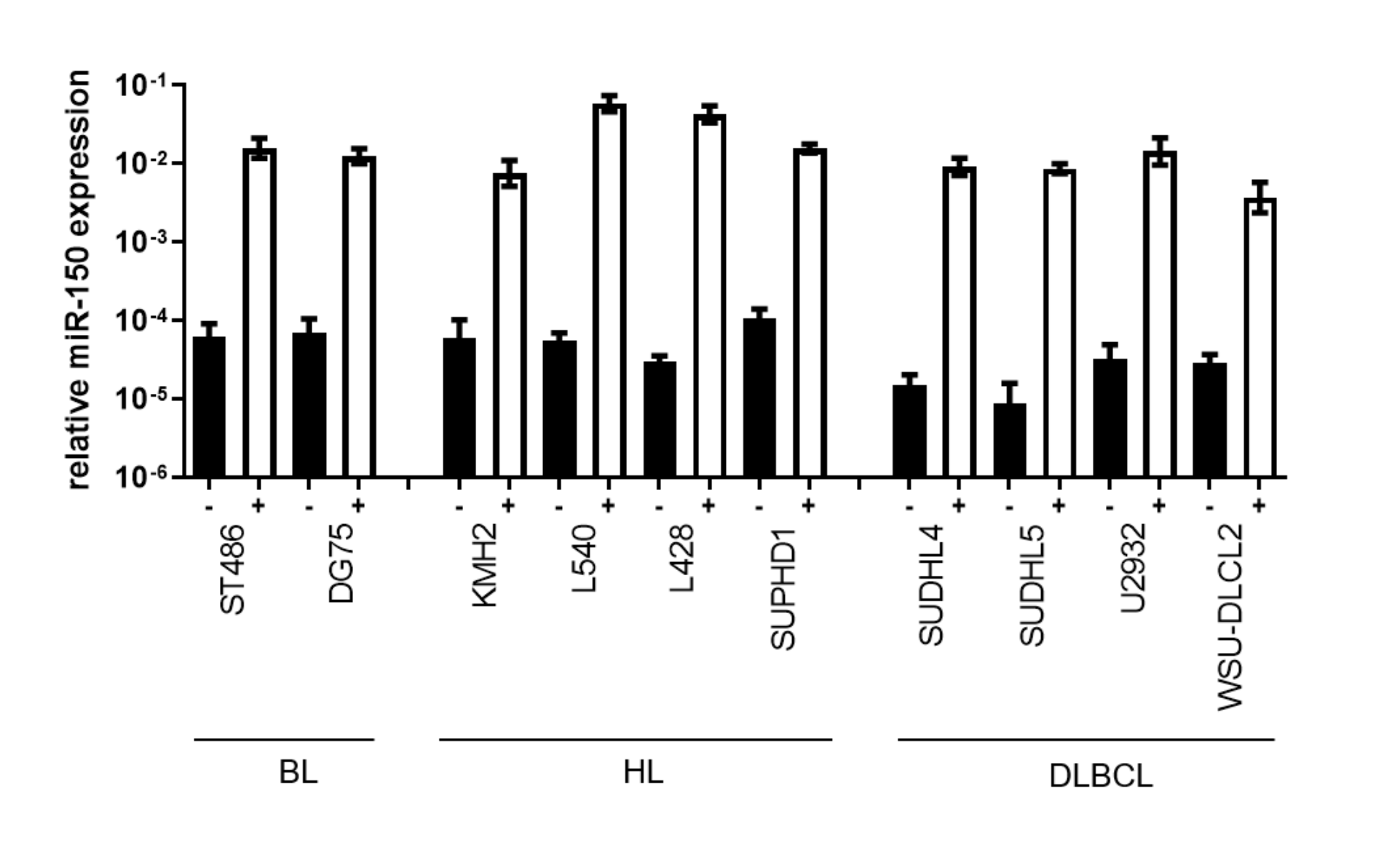

Supplement: Supplementary file 1 [file genes-13-00227-s001.zip › Figure S5.tif]

A

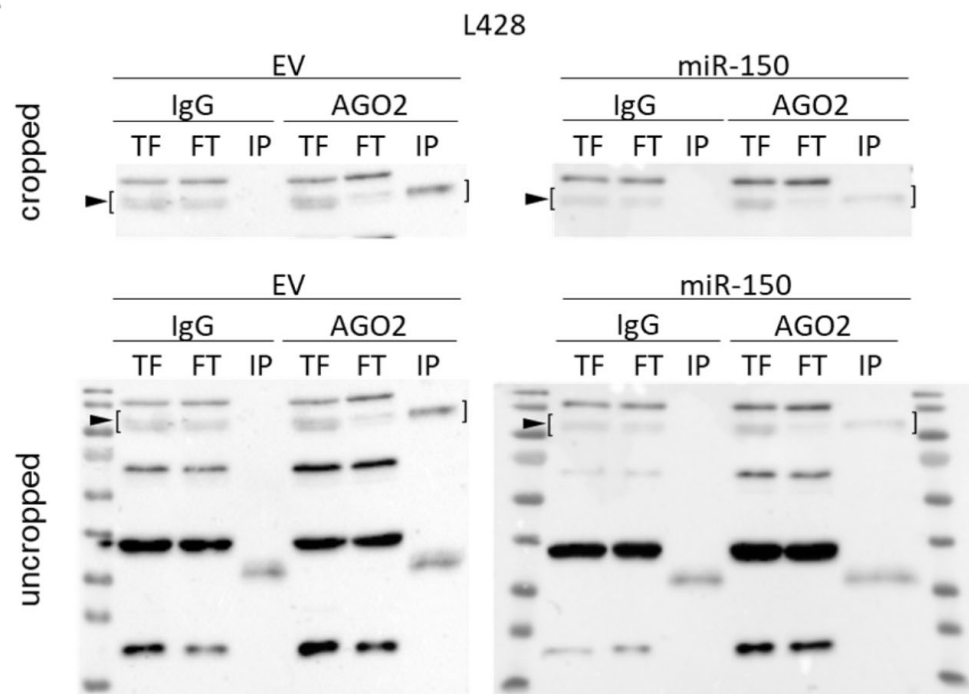

B

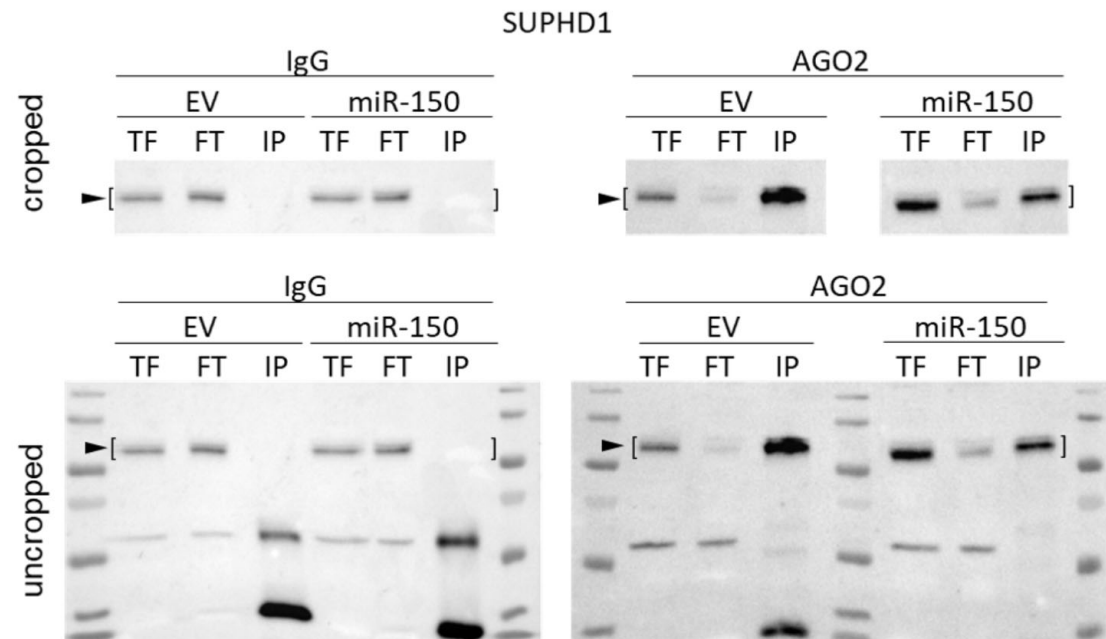

C

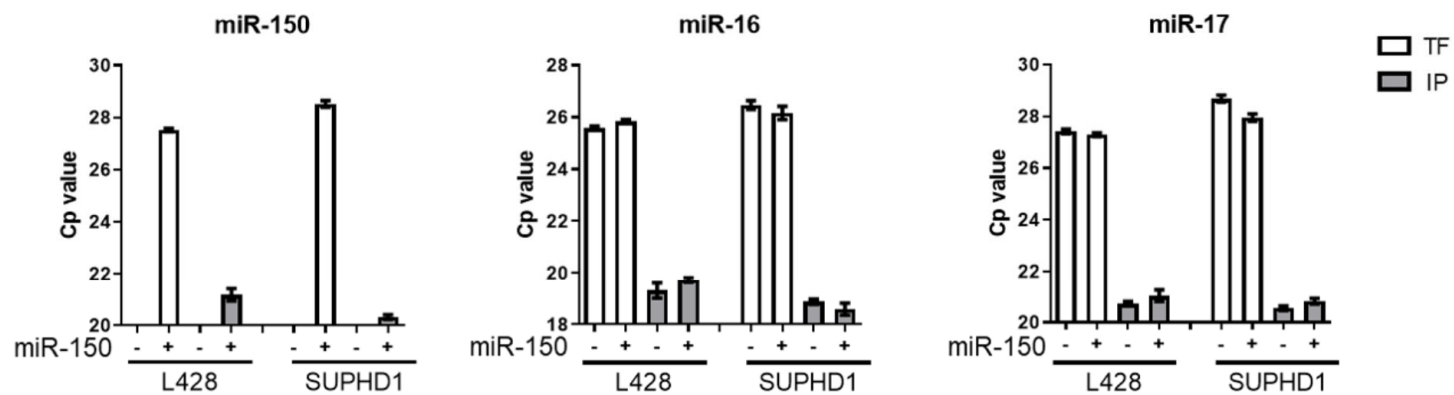

Supplement: Supplementary file 1 [file genes-13-00227-s001.zip › Figure S6.pdf]

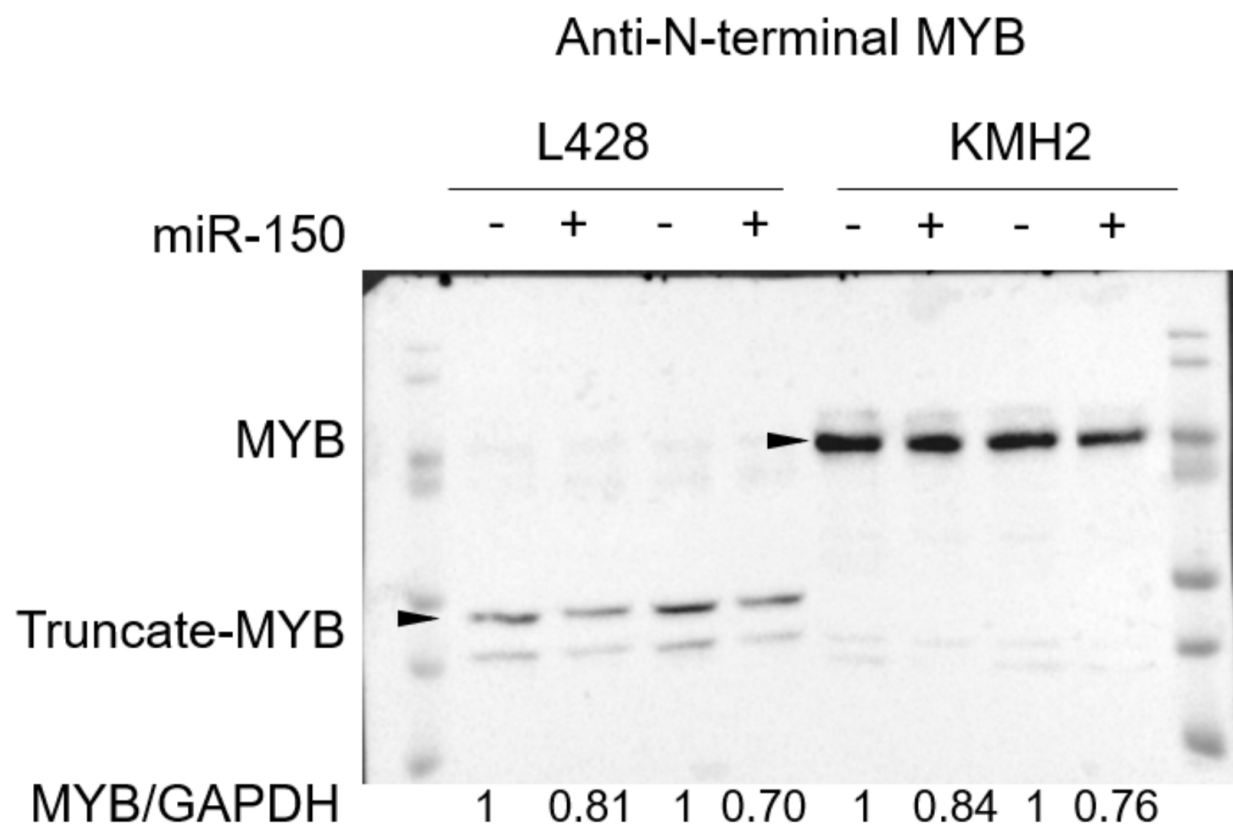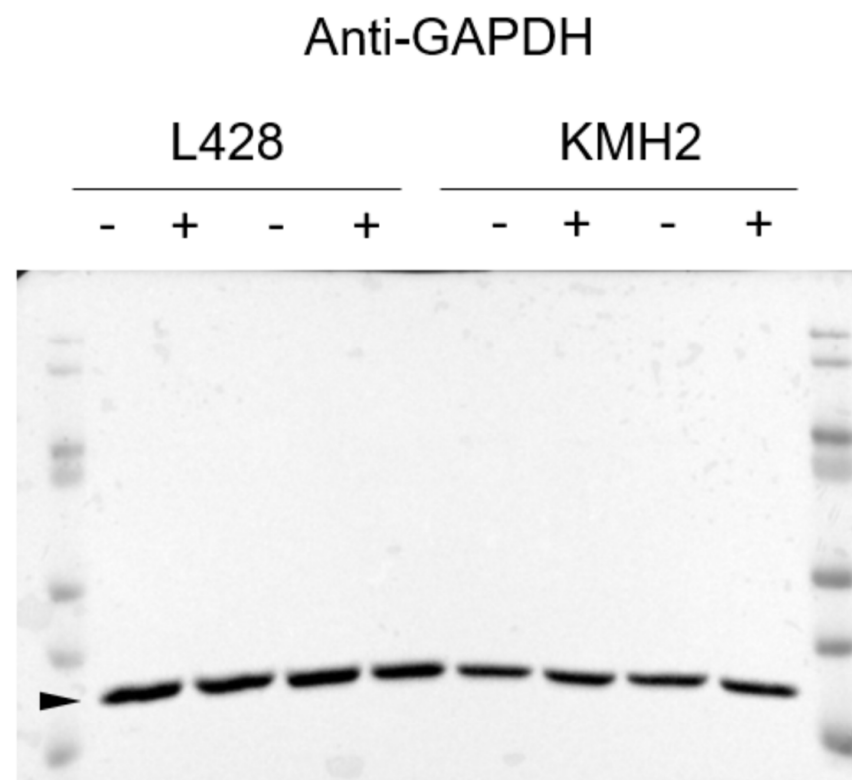

Supplement: Supplementary file 1 [file genes-13-00227-s001.zip › Figure S7.pdf]

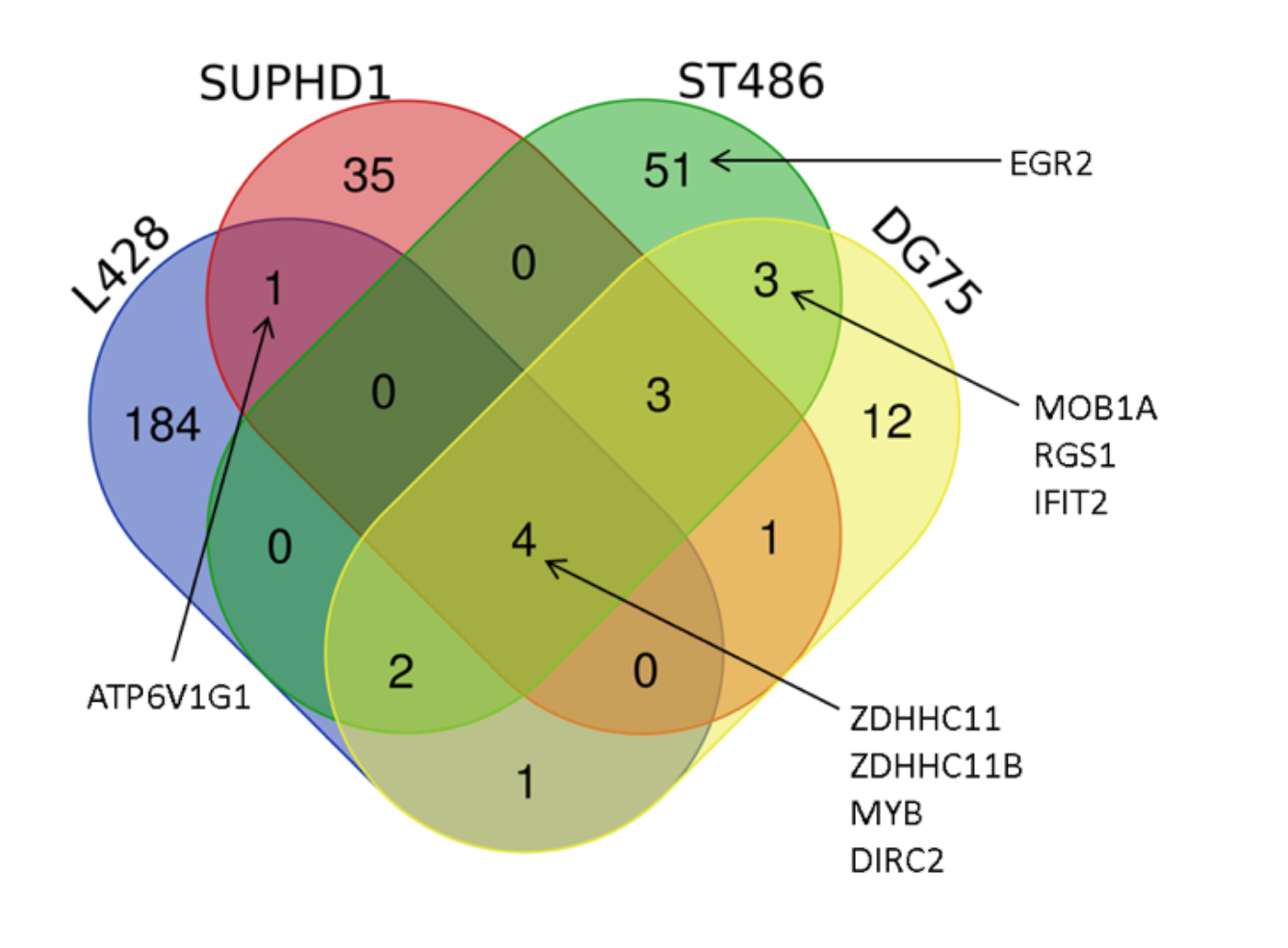

Supplement: Supplementary file 1 [file genes-13-00227-s001.zip › Figure S8.tif]
